# Dynamic Interplay of Nonlocal Recombination Pathways in Quantum Emitters in Hexagonal Boron Nitride

**DOI:** 10.1021/acs.jpcc.4c07147

**Published:** 2025-01-16

**Authors:** Enrique A. Mejia, John M. Woods, Ashok Adhikari, Charanjot Singh, Takashi Taniguchi, Kenji Watanabe, Valentina Bisogni, Zdeněk Sofer, Jonathan Pelliciari, Gabriele Grosso

**Affiliations:** † Photonics Initiative, Advanced Science Research Center, City University of New York, New York, New York 10031, United States; ‡ Research Center for Electronic and Optical Materials, National Institute for Materials Science, 1-1 Namiki, Tsukuba 305-0044, Japan; § National Synchrotron Light Source II, Brookhaven National Laboratory, Upton, New York 11973, United States; ∥ Department of Inorganic Chemistry, University of Chemistry and Technology Prague, Technická 5, Prague 6 166 28, Czech Republic; ⊥ Physics Program, Graduate Center, City University of New York, New York, New York 10016, United States

## Abstract

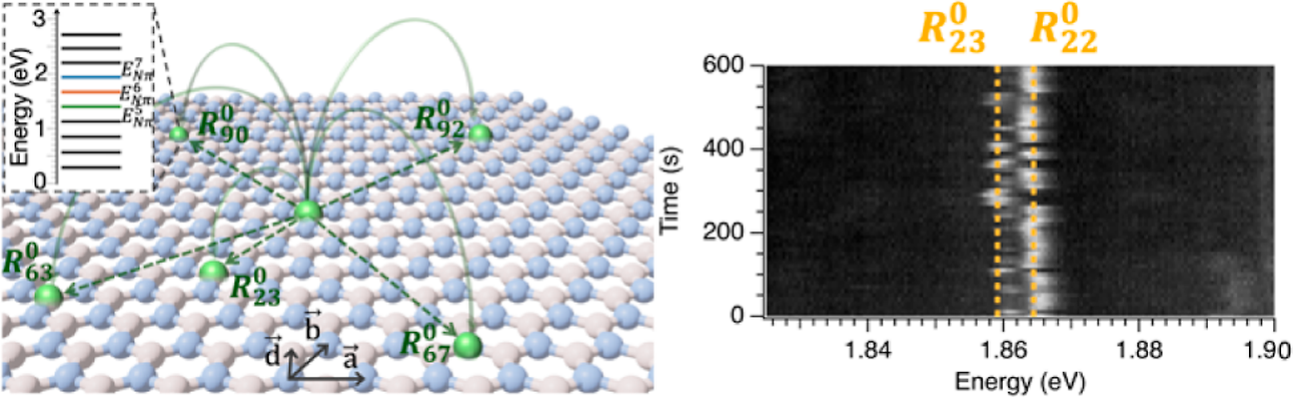

Optically active defects in wide bandgap materials play a central role in several emerging applications in quantum information and sensing as they allow for manipulating and harvesting the internal degrees of freedom of single electrons with optical means. Interactions among defect states and with the surrounding environment represent a crucial feature for sensing but can severely hamper the coherence of the quantum states and prevent an efficient integration with photonic architectures due to unpredictable spectral instability. Understanding and controlling defect interactions would mitigate the effects of spectral instabilities and enable quantum applications based on long-range interactions. Here, we investigate the photoluminescence spectral dynamics of quantum emitters in defective hexagonal boron nitride (hBN), a material whose emission spectrum notoriously displays spectral wandering and diffusion, and we identify several optical transitions with discrete energy jumps. We associate the spectral jumps with the interplay amid competing recombination pathways available to the defect states in a process like donor–acceptor-pairs (DAP). The discrete spectral jumps observed in the emission spectrum of hBN arise from interactions between the harmonic states of nitrogen π orbitals of delocalized defects, and their energies can be ascribed to a DAP-like transition sequence. Our results allow mapping of the defect geometry in an hBN lattice, setting the basis for mitigating the effects of spectral jumping in this platform and paving the way toward using the long-range interaction of defect ensembles for quantum technology.

## Introduction

Solid-state quantum emitters like quantum dots and color centers are viable systems for a variety of applications in integrated photonics and quantum technology due to their use as single-photon sources, spin qubits, and sensors.^1,2^ Moreover, their straightforward integration onto complex photonic architectures makes them advantageous for the development of on-chip quantum photonic technology.^3,4^ The mutual and environmental interactions of such emitters provide several opportunities for enhanced quantum sensing^5,6^ while presenting challenges for their application in quantum simulators^7^ and qubits.^8^ Environmental interactions with electrons, ions, or phonons are associated with spectral instabilities, namely, the random changes of energy and intensity of the photoluminescence emission. Spectral instabilities in solid-state emitters present several practical drawbacks by limiting photon indistinguishability,^9^ quantum coherence,^10^ sensitivity,^5,6^ and effective coupling with high-quality optical resonators and waveguides.^11,12^ The amplitude and time scales of the dynamics of the spectral emission of solid-state emitters can be affected by several underlying physical mechanisms such as variations in temperature,^13^ magnetic,^14^ and electric fields.^15,16^ Among these effects, spectral diffusion is common across many materials and appears as the wandering of the emission wavelength around a central value.^17,18^ Spectral diffusion linked to an electromagnetic environment evolving faster than the radiative lifetime can induce severe broadening of emission lines which can be detrimental to many applications.^10,19,20^ Spectral blinking refers to the dynamic switching on and off of the emission, and in some systems has been explained with the presence of long-lived dark states within the energy landscape of the emitter.^21^ Spectral jumps can also occur in solid-state emitters through the random swapping of the emission energy between two or more discrete values.^22,23^ Several experimental schemes have been proposed to mitigate the effects of spectral instabilities, including the use of tailored sequences of optical pulses,^24^ anti-Stokes excitation,^25^ capping or isolating layers,^26,27^ specific growth techniques,^28,29^ and postgrowth methods such as high-temperature annealing in different gas environments.^30^ Nonetheless, fluctuations in the optical response of defect-based emitters can also provide a valuable opportunity to shed light on hidden physical processes such as long-range dipole interactions and help reconstruct the recombination dynamics within defective states.

The emergence of defect-based quantum emitters in hexagonal boron nitride (hBN) has spurred interest due to their room temperature operation,^31,32^ advantageous optical properties,^33^ and promising spin structures.^34−39^ However, defect emission in hBN shares the same limitations due to spectral instabilities with other materials such as NV centers in diamond,^40,41^ SiN,^42^ and several quantum dot platforms.^18,43−45^ The challenges associated with spectral instabilities in hBN are aggravated by the uncertainty regarding the microscopic origin of hBN emitters that prevents the development of methods to target and correct for the physical mechanisms underlying their photoluminescence (PL) emission.^46,47^ Emitters in hBN span a large portion of the spectrum, from the infrared to the UV,^31,48,49^ suggesting that quantum emitters could have different microscopic origins. However, only a few structures have been clearly identified, such as the *V*_B_^–^ defect, responsible for emission at around 800 nm.^50^ Other studies have suggested the involvement of carbon^46^ or oxygen^51^ impurities in the defective structures responsible for optical quantum emission. Recently, experimental evidence showed that most of the quantum emission in the visible range in defective hBN samples can be ascribed to nonlocal radiative recombination similar to donor–acceptor-pair (DAP) processes^52^ whose fundamental energies are associated with harmonic states stemming from the π* orbitals of nitrogen.^47^ The uncovering of these elementary excitations of quantum emitters in hBN and their recombination processes has opened new avenues to model hBN emitters and explain associated phenomena that are still under debate including their mutual interaction and the resulting spectral instabilities.

Here, we investigate the low-temperature PL spectra of defective hBN samples prepared in two distinct ways. The first method creates defects in a bottom-up process by flux growth synthesis in bulk crystals, which are then exfoliated and annealed to activate defect-based emission. The second is a high-quality pristine hBN^53^ that is exfoliated, irradiated with argon plasma, and then annealed at high temperature to generate a high density of active defects in a top-down fashion^54^ (see Methods). We refer to these as sample 1 and sample 2, respectively. By analyzing the PL emission spectra of both samples with a model that includes the harmonic states at the N-π* orbitals and a DAP-like nonlocal recombination process,^47,55^ we show that the spectral jumps in both hBN samples stem from the interplay between nonlocal recombination processes among neighboring defective sites. We observe spectral jumps following a discrete pattern that can be well reproduced by a DAP-like process. From a microscopic perspective, this observation indicates that the change in the emission energy is due to the switch of the dipole transition from multiple sites. Our approach allows us to reconstruct the defective configuration of the sample and formulate hypotheses regarding the electronic dynamics underlying single photon emission.

## Methods

### Sample Growth and Preparation

Sample 1 was prepared from molten metal flux in an ammonia atmosphere in a vacuum tight bottom loading furnace. The growth was performed from 100 g of iron (99.98%, 2–4 mm, Alfa Aesar) mixed with 6 g of hBN powder (99.5%, 30 μm). The mixture was loaded in a furnace, which was flushed with argon under a vacuum and heated to 1000 °C. Subsequently, the furnace was filled with a mixture of ammonia and hydrogen. The ratio of gases was 50 sccm of NH_3_ and 100 sccm of H_2_. The furnace was then heated to 1600 °C using a heating rate of 5 °C/min and after 24 h cooled to 1000 °C using a cooling rate of 1 °C/min and subsequently freely cooled to room temperature overnight. Formed hBN crystals were mechanically removed from the metal surface and subsequently exfoliated on a Si/SiO_2_ substrate. Sample 2 was prepared by exfoliating highly pure hBN^53^ on a Si/SiO_2_ substrate, followed by a treatment based on reactive ion etching (RIE) in a 50 mTorr argon environment with a flow rate of 30 sccm using 50 W of forward power for 5 min. Both treatments of hBN are subsequently annealed in a nitrogen environment for 2 h at 950 C.

### PL Measurements

**Figure 1:**
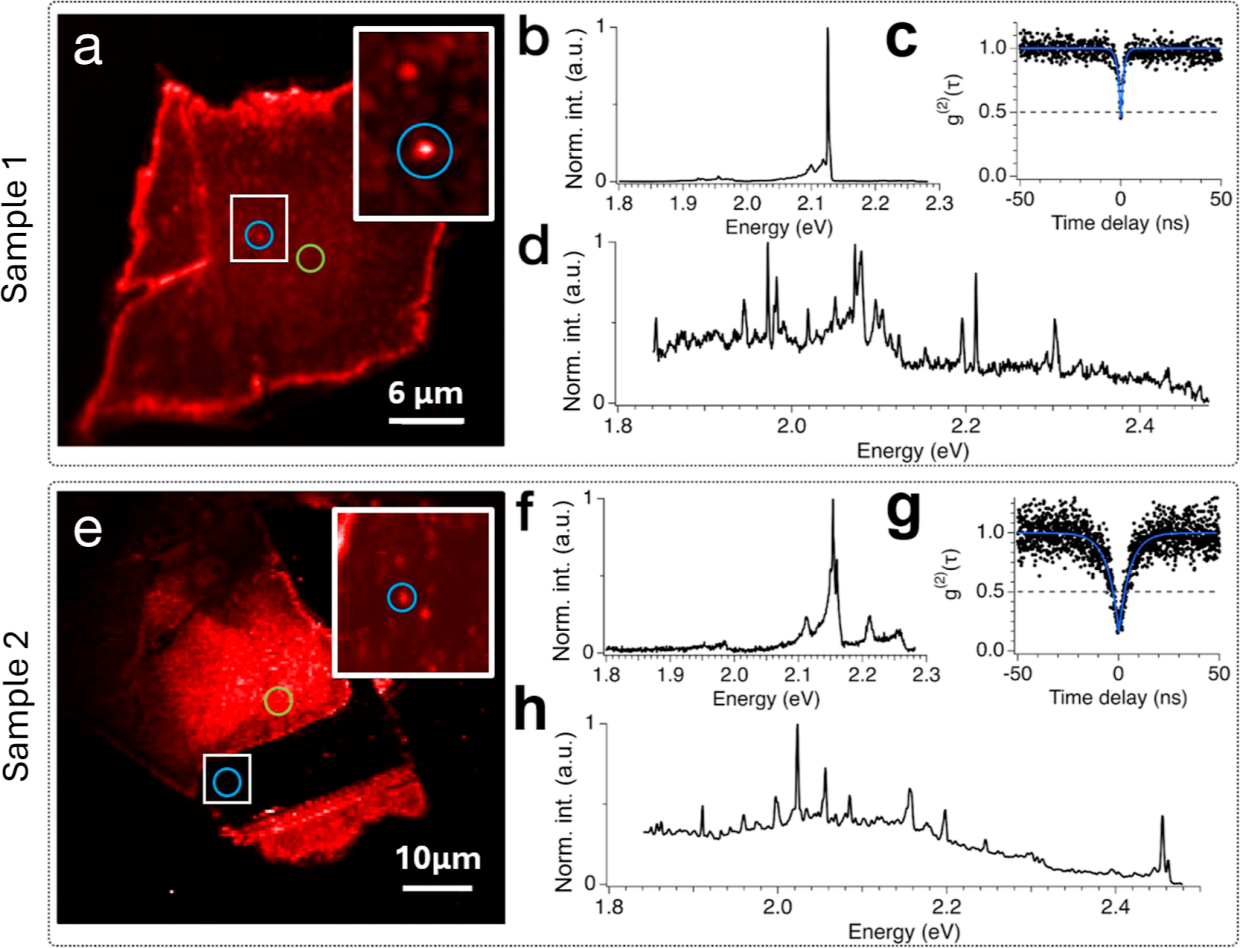
Characterization of defective hBN samples. (a) PL map of sample 1 with blue and green circles corresponding to the position where the spectra in Figure 1b,d are taken, respectively. (b) Emission spectrum taken from a bright, isolated emitter (indicated by the blue circle in Figure 1a), showing a sharp ZPL emission followed by low-energy phonon sidebands. (c) Measurement of the second-order autocorrelation function (g^(2)^(t)) of the emitter whose spectrum is shown in Figure 1b. Antibunching with g^(2)^(0) = 0.45 confirms the single photon emission (experimental data in black, fit line in blue). (d) Emission spectrum taken in a region with high density of emitters (green circle in Figure 1a) showing multiple sharp peaks. (e) PL map of sample 2 with blue and green circles corresponding to the position where the spectra in Figure 1f,h are taken, respectively. (f) Emission spectrum taken from a bright, isolated emitter (indicated by the blue circle in Figure 1e), showing a sharp ZPL emission followed by low-energy phonon sidebands. (g) Measurement of the second order autocorrelation function (g^(2)^(t)) of the emitter whose spectrum is shown in Figure 1f. Antibunching with g^(2)^(0) = 0.16 confirms the single photon emission. (h) Emission spectrum taken in a region with high density of emitters (green circle in Figure 1e) showing multiple sharp peaks.

**Figure 2:**
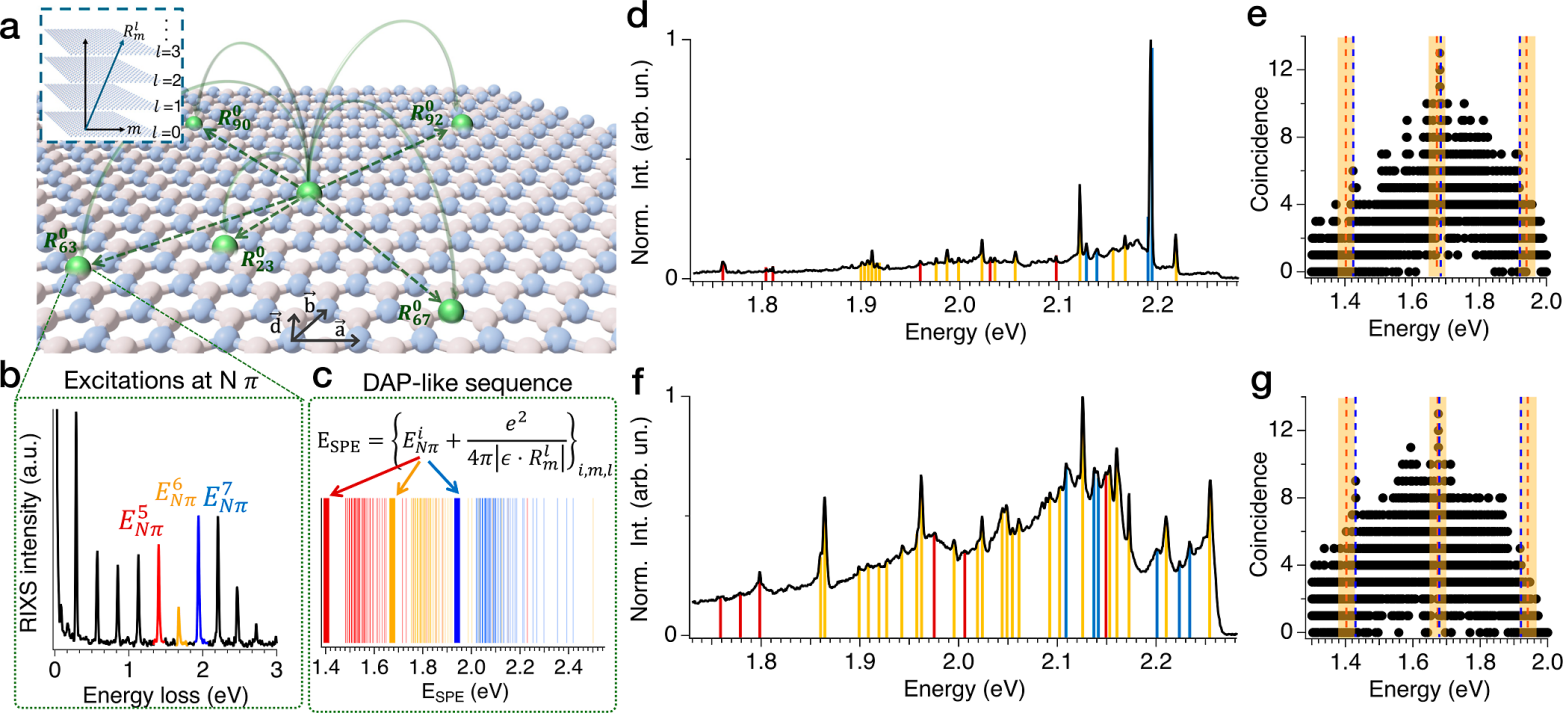
DAP-like recombination in defective hBN samples. (a) Cartoon illustrates the principles of DAP-like recombination processes for single layer hBN hosting DAP-like pairs. Inset describes the definition of *R*_m_^*l*^, where m is an ordered index starting from the closest transitions and working toward larger jumps in the in-plane direction, and *l* is the number of layers among which the transition occurs. (b) RIXS spectrum of a plasma-treated defective hBN measured at the N K pre edge in resonance with the nitrogen π* orbitals showing harmonic series of states. Harmonic states corresponding to *E*_Nπ_^5^ = 1.403 eV, *E*_Nπ_^6^ = 1.676 eV, and *E*_Nπ_^7^ = 1.941 eV are highlighted in colors used to define the different DAP-like sequences associated with them. (c) DAP-like sequence computed from the model of the SPE emission pattern (*E*_SPE_) from the harmonic states *E*_Nπ_^5^, *E*_Nπ_^6^, and *E*_Nπ_^7^. (d) Composite PL spectrum of sample 1 produced by combining 60 spectra taken in the same location of the flake. The breakdown of the time evolution of the spectrum is shown in Figure 3a. Vertical lines indicate the matched DAP transitions to the fit. (e) Results of the DAP fitting of the peak energies extracted from the composite spectrum of Figure 2d showing the coincidence counts among the sequence of the experimental peaks and the DAP model for an hBN lattice as a function of the energy of the fundamental transition. Three local maxima (highlighted by vertical blue dashed lines) at *E*_Nπ_^5^ = 1.424 eV, *E*_Nπ_^6^ = 1.683 eV, and *E*_Nπ_^7^ = 1.916 eV emerge in the neighborhood of the RIXS values (indicated by vertical red dashed lines). The matched energies of the experimental data are indicated by dotted vertical lines in Figure 2b. (f) Composite spectrum of sample 2 built by combining 60 spectra taken in the same location of the flake. The breakdown of the time evolution of the spectrum is shown in Figure 4a. (g) Results of the DAP fitting of the peak energies extracted from the composite spectrum of Figure 2d showing the coincidence counts among the sequence of the experimental peaks and the DAP model for an hBN lattice as a function of the energy of the fundamental transition. Three local maxima (highlighted by vertical blue dashed lines) are found in the neighborhood of RIXS values for *E*_Nπ_^5^, *E*_Nπ_^6^, and *E*_Nπ_^7^ (indicated by vertical red dashed lines). The matched energies of the experimental spectrum are indicated by overlaid vertical lines in Figure 2f. Shaded regions in (e, g) indicate the interval of confidence for the energy of the harmonic states as measured from RIXS, corresponding to ±20 meV, roughly corresponding to the resolution of RIXS measurements.

Time-dependent PL measurements and mapping are performed in a home-built micro-PL setup coupled to a closed-cycle cryostat (*T* = 8K). Spectra in Figure 2, as well as in Figure 1b,f, are produced by exciting hBN with a continuous wave (CW) 532 nm laser in a reflection geometry with an incident power of 250 μW and a laser spot with a diameter of 2 μm on the sample. The 532 nm laser signal is removed from the collection path using a 550 nm long-pass filter. To capture additional defective emission at higher energy, spectra in Figure 1d,h are taken using excitation from a CW 473 nm laser, and laser signal is filtered using a 500 nm long-pass filter. Due to the lower efficiency of the optical setup when the samples are excited with the 473 nm laser, all other measurements are taken by using the 532 nm laser. g^(2)^(t) measurements are performed in a home-built Hannbury–Brown–Twiss (HBT) interferometer. Time-dependent spectra are taken on a spectrometer with a grating of 150 lines per millimeter paired with an electron multiplied charge coupled device (EMCCD).

### RIXS Data

RIXS measurements were performed at the 2-ID (SIX) beamline at NSLS-II, Brookhaven National Laboratory. All of the samples are aligned with the surface normal (001) parallel to the scattering plane. The spectrometer arm is positioned at a fixed scattering angle of 90°. The incident light is σ polarized. The energy resolution is about 20 meV full width at half-maximum (FWHM) at the N–K edge. All of the RIXS measurements are performed at 300 K.

## Results and Discussion

We initially characterize the emission of the hBN samples by recording the PL maps of the whole flakes and the spectral emission in different regions. Figure 1a shows the PL map of sample 1, which is characterized by an intense sub-band emission due to the presence of a large density of optically active defects. However, in some locations, like the one highlighted by the blue circle, single emitters are much stronger than the background, exhibiting a spectrum with a single zero-phonon-line (ZPL), as illustrated in Figure 1b. Measurement of the second-order autocorrelation function (g^(2)^(0)) returns an antibunching of 0.45, below the threshold of 0.5, confirming the quantum nature of the emission. Autocorrelation data are fitted with the function g^(2)^(t)=1-Ae^-t/τ^, where τ is the lifetime of the excited state, and *A* is the fitting parameter. Other locations across flakes of sample 1 host a high density of emitters, and the spectra (typified by Figure 1d collected from the green circle in Figure 1a) display many sharp peaks emerging from the background due to the presence of a larger density of active defects. As discussed below, this sequence of lines can be matched within a DAP-like model that considers several delocalized recombination processes. Similar PL properties are observed in sample 2 (Figure 1e–h). As with sample 1, it is possible to isolate single ZPLs from localized emitters whose spectrum includes a single dominant peak (Figure 1f) and regions of higher defect density with multiple peaks (Figure 1h). Second-order autocorrelation measurements confirm the presence of quantum emitters in this hBN sample with an antibunching of g^(2)^(0) = 0.16.

To rationalize the complex spectrum from sample regions with high density of defects, we follow the approach used in ref (47,55) and the phenomenological model therein developed. Resonant inelastic X-ray scattering (RIXS) performed in highly defective hBN samples revealed the presence of a harmonic series of electronic states stemming from the π* orbitals of nitrogen, which are strongly correlated to the defect-based quantum emission. This correlation becomes evident when the peaks in the PL emission spectrum are framed within a DAP series whose fundamental energies are given by the harmonic states at the N-π* orbitals. In ref (47) it was proven that electrons excited to the harmonic states at the N-π* orbitals can recombine nonlocally with other electronic states available in different lattice sites, generating quantum emission (Supporting Information note 1). Figure 2a–c illustrate the basic principles of this model that predicts an emission spectrum that follows the sequence , where *E*_Nπ_^*i*^ is the series of energies of the harmonic states at the *N*-π* orbitals measure by RIXS (shown in Figure 2b) indexed by the integer number *i*, ε is the dielectric tensor of hBN, and  are vectors connecting all possible atomic sites within the hBN lattice. These vectors, indexed by the integer number d that indicates the shell number of the Coulomb DAP pair,^56^ are calculated starting from the lattice vectors in a 3D hBN structure with . Examples of the  vectors within a single hBN layer and the nonlocal recombination processes are depicted in the cartoon of Figure 2a. To parametrize the DAP shells and include the information regarding the transitions occurring among different layers, we will refer to  vectors with the label *R*_m_^*l*^, where m is an ordered index starting from closest transitions and working toward larger jumps in the in-plane direction, and *l* is the number of layers among which the transition occurs (see inset of Figure 2a and Supporting Information note 2 for more details on this notation). To confirm the validity of this model, we perform a fit of the energies of the peaks extracted from the PL spectra in both hBN samples with the energy sequences *E*_SPE_ (Figure 2c) using the algorithm described in ref (55). This statistical analysis compares two discrete sequences, the energies of the peaks in the PL spectra, and an ideal DAP sequence, *E*_SPE_, and returns the number of coincident peaks among them as a function of the fundamental DAP energy used as a fitting parameter. The coincidence represents the fidelity of the DAP series (and its parameters) to the experimental data. As discussed in ref (55), this approach identifies nonlocal recombination processes from PL spectra and has been proven to be effective to understand the emission spectra of defective hBN, confirming the results of previous experimental works.^47,52^ We note that this model explains most of the emission peaks in the visible spectrum of defective hBN but does not preclude the presence of other active defects whose emission has a different microscopic origin and is unrelated to the N-π* orbitals.

To include the spectral instability in our investigation of the hBN emission, we perform the DAP analysis on composite spectra that includes the spectral dynamics over the course of 600 seconds on both samples 1 and 2. To do so, we continually record the spectrum from the same position of an hBN flake with an integration time of 10 seconds. Then, we combined all of these spectra by summing them and normalizing the intensity. All PL spectra in this work have been measured at *T* = 8K. Figure 2d,f are the combined spectra from samples 1 and 2, respectively. The corresponding coincidence plots resulting from the DAP fit are depicted in Figure 2e,g. Here, we have considered matches to be found when the energies of the PL peak and a DAP transition are within a restrictive coincidence window of 0.6 meV, which is approximately the average broadening of the peaks observed in the spectra. In both samples, we identify three fundamental energies for the DAP process (characterized by a local maximum of the coincidence plots) that are in excellent agreement with the harmonic states measured by RIXS^47^ and the values measured in previous works on DAP processes in hBN.^52^ We find *E*_Nπ_^5^ = 1.424 ± 0.001 eV, *E*_Nπ_^6^ = 1.683 ± 0.002 eV, and *E*_Nπ_^7^ = 1.916 ± 0.001 eV for sample 1, and *E*_Nπ_^5^ = 1.430 ± 0.003 eV, *E*_Nπ_^6^ = 1.676 ± 0.003 eV, and *E*_Nπ_^7^ = 1.918 ± 0.001 eV for sample 2. The peaks in the composite spectra that are matched with the DAP transitions are marked by overlaid vertical bars in Figure 2d,f. Corresponding values of *R*_m_^*l*^ can be found in Supporting Information note 5. The values of the dielectric and the lattice vectors used to generate the sequence *E*_hBN_ that fit the data for samples 1 and 2 are reported in Tables S1 and S2 in Supporting Information note 4 along with the full list of the DAP transitions and the corresponding shell values. After performing the initial DAP fit of the data and confirming the presence of fundamental energies for the DAP process comparable with the harmonic states at the N-π* orbitals, we perform a direct search around these values by fine-tuning the lattice and dielectric constants and performing fits to maximize the coincidence.^55^ This addition fitting step is included to account for the possible local variations of the environment in different hBN samples and flakes.^56^ Through the direct search process, lattice parameters are found to be consistent across samples with values of *a* = 2.520 Å and *c* = 6.673 Å. For the sample 1, we find ϵ_∥_ = 6.999 and ϵ_⊥_ = 3.685, while in sample 1, we find ϵ_∥_ = 6.916 and ϵ_⊥_ = 3.741. Each of these pairs of values are in good agreement with the literature on hBN,^57^ and small variations can be expected for samples grown with different methods. We note that in our experiments, we only detect the harmonic states *E*_Nπ_^5^, *E*_Nπ_^6^, and *E*_Nπ_^7^, whose energy is within reach of our experimental optical setup.

**Figure 3:**
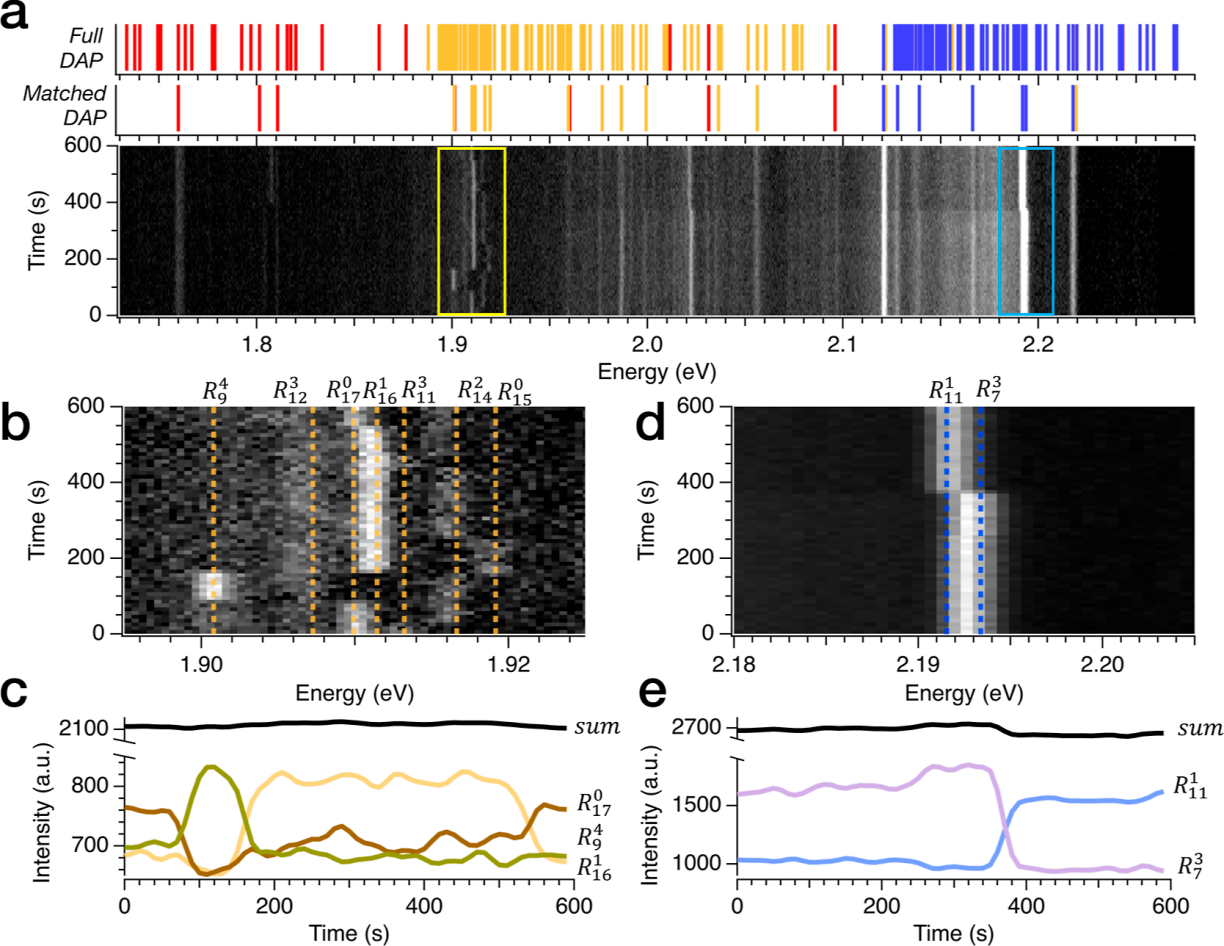
Signature of the interplay among nonlocal recombination pathways in the spectral dynamics of sample 1. (a) False color map is the normalized time trace of the spectral dynamics of sample 1. It is composed of spectra captured every 10 seconds over 600 seconds in the same location of the hBN flake. Top panel shows the representative DAP sequence generated with the fitting parameters extracted from the analysis in Figure 2e. The lattice parameters are a = 2.52 Å, c = 6.673 Å, ϵ∥ = 6.999, and ϵ⊥ = 3.685. The energy of the fundamental energy of the harmonic states are *E*_*Nπ*_^5^*=* 1.424 eV, *E*_Nπ_^6^ = 1.683 eV, and *E*_Nπ_^7^ = 1.916 eV, highlighted in red, orange, and blue, respectively. Bottom panel shows the line of the DAP sequences that found matches in the emission spectrum. (b) Zoom of time-dependent spectra corresponding to the portion indicated with the yellow box in (a). Note how peaks associated with *R*_9_^4^, *R*_17_^0^, and *R*_16_^1^ corresponding to *E*_Nπ_^6^ = 1.683 eV appear to be correlated in time with one another while lines corresponding to *R*_11_^3^, *R*_14_^2^, and *R*_15_^0^ corresponding to *E*_Nπ_^6^ = 1.683 eV appear to be similarly correlated (Figure S3). (c) Intensity of the peaks *R*_9_^4^, *R*_17_^0^, and *R*_16_^1^ as a function of time. The almost constant sum of the intensity confirms the correlation among these states. (d) Zoom of time-dependent spectra corresponding to the portion indicated with the blue box in (a). Peak associated with *R*_7_^3^ switch to a lower-energy state that correspond to *R*_11_^1^. Both states stem from the fundamental energy *E*_Nπ_^7^ = 1.916 eV. (e) Intensity of the peaks *R*_7_^3^ and *R*_11_^1^ as a function of time. The almost constant sum of the intensity confirms the correlation among these states.

Having confirmed the presence of delocalized recombination processes in both hBN samples, we now discuss the spectral dynamics of their emission with a particular focus on the spectral jumps that are frequently observed in hBN.^27,58^Figure 3a is the time-dependent series of spectra recorded in sample 1, and when summed, it generates Figure 2d. Here, we can appreciate the dynamics of the emission spectrum on a time scale of 10 seconds, corresponding to the integration time of each spectrum. In sample 1, the overall emission is rather stable over time, but a few lines present clear spectral jumps among different energy states, as in the two spectral regions highlighted by the yellow and cyan boxes. The zoomed-in plot in the energy region around 1.9 eV, shown in Figure 3b, reveals complex dynamics with the emission jumping between multiple states. We are able to associate these peaks to the nonlocal recombination processes of the *E*_Nπ_^6^ state (1.683 eV) corresponding to *R*_m_^l^ = *R*_15_^0^, *R*_14_^2^, *R*_11_^3^, *R*_16_^1^, *R*_17_^0^, *R*_12_^3^, *R*_9_^4^ (shell values are in Supporting Information Table S1). We identify a strong correlation among states corresponding to *R*_m_^l^ = *R*_16_^1^, *R*_17_^0^, *R*_9_^4^, as manifested by their intensity traces (Figure 3c). The sum of their intensity is almost constant over time suggesting that this emission stems from the same underlying process that can be associated with the delocalized recombination process of the state *E*_Nπ_^6^ = 1.683 eV among different defect locations in the atomic lattice. Another set of correlated states can be observed in the trace of Figure 3b with an interplay present between less intense lines attributed to *R*_m_^l^ = *R*_15_^0^, *R*_14_^2^, *R*_11_^3^, while a faint but consistent line *R*_m_^l^ = *R*_12_^3^ remains present for the entirety of acquisition. Discussion on the correlation between states associated with transitions at *R*_m_^l^ = *R*_15_^0^, *R*_14_^2^, *R*_11_^3^ can be found in Figure S3. A similar dynamic with discrete spectral jumps is observed in other regions of the spectrum, such as the one around 2.2 eV (blue box in Figure 3a) and shown in the zoomed-in plot of Figure 3d. Here, we see that at around 400 seconds, the main emission undergoes a clear jump among two states that can be associated with *R*_m_^l^ = *R*_11_^1^ and *R*_7_^3^ of the state *E*_Nπ_^7^ = 1.918 eV. The intensities of the time traces are clearly correlated (Figure 3e), indicating that the interplay of these two defective sites is the underlying mechanism of the spectral jumps in this part of the spectrum. It should be noted that two peaks at around 2.021eV experiences the same dynamics to the peak associated with the states *R*_11_^1^ and *R*_7_^3^ (traces and discussion in Supporting Information note 7 and Figure S4). The energy detuning among these two emission features (approximately 170 meV) is compatible with the phonon sidebands of quantum emitters previously observed in hBN.^31,33,36^ Therefore, we ascribed these peaks to phonon replicas of the states *R*_11_^1^ and *R*_7_^3^, and we choose to remove these lines from our DAP fit.

**Figure 4:**
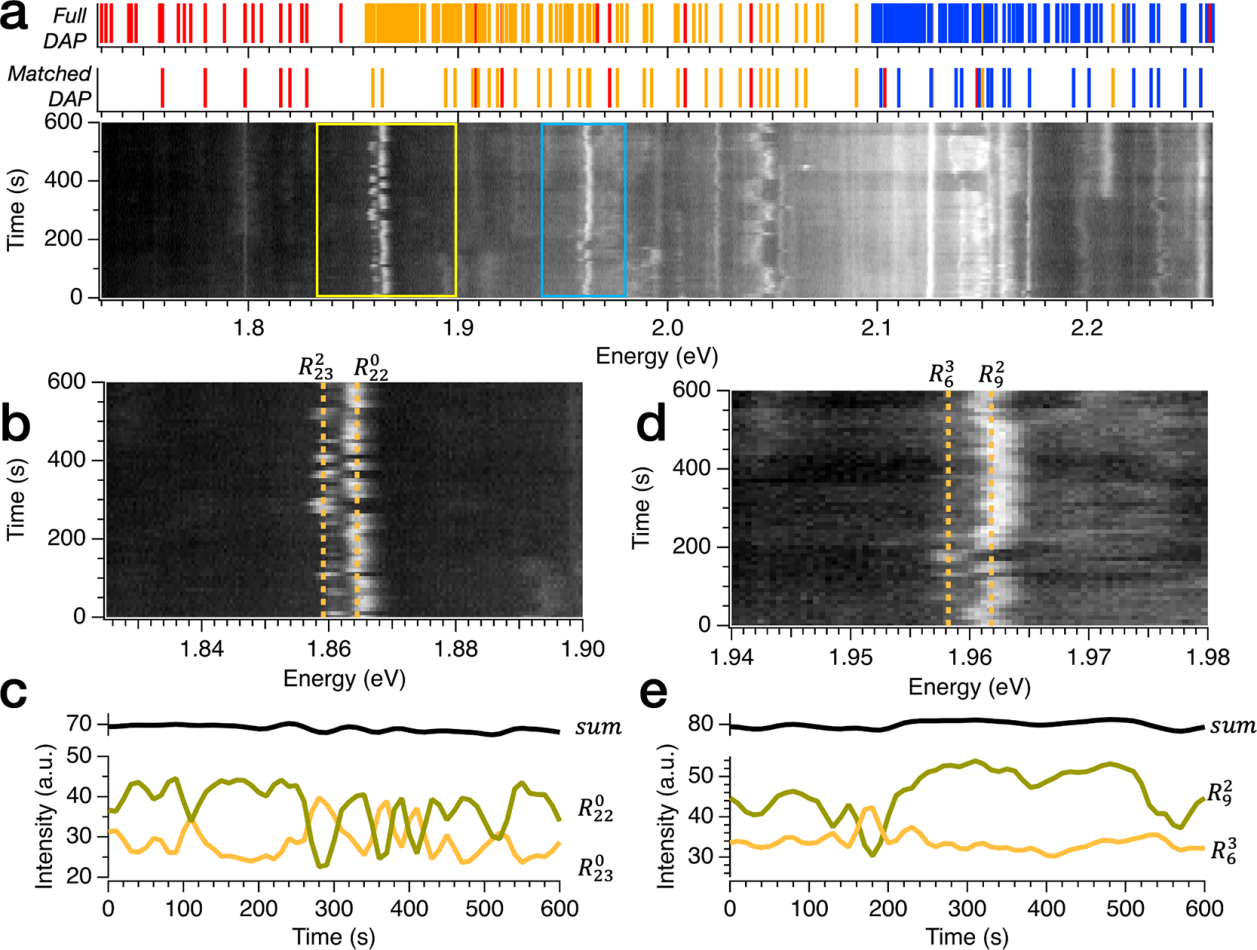
Signature of the interplay among nonlocal recombination pathways in the spectral dynamics of sample 2. (a) Plot is the normalized time trace of the spectral dynamics of sample 2. It is made with spectra captured every 10 seconds over 600 seconds in the same location of the flake. The top panel shows the representative DAP sequence generated with the fitting parameters extracted from the analysis in Figure 2g. The lattice parameters are a = 2.52 Å, c = 6.673 Å, ϵ∥ = 6.916, and ϵ⊥ = 3.741. The energy of the fundamental energy of the harmonic states are *E*_*Nπ*_^5^ = 1.430 eV, *E*_Nπ_^6^ = 1.676 eV, and *E*_Nπ_^7^ = 1.917 eV, highlighted in red, orange, and blue, respectively. The bottom panel shows the line of the DAP sequences that found matches in the emission spectrum. (b) Zoom of time-dependent spectra corresponding to the portion indicated with the yellow box in a. Note how peaks associated with *R*_23_^0^ and *R*_22_^0^ corresponding to *E*_Nπ_^6^ appear to be correlated in time with one another. (c) Intensity of the peaks *R*_23_^0^ and *R*_22_^0^ as a function of time. The almost constant sum of the intensity confirms the correlation among these states. (d) Zoom of time-dependent spectra corresponding to the portion indicated with the blue box in (a). Peaks associated with *R*_6_^3^ and *R*_9_^2^ corresponding to *E*_Nπ_^7^ appear to be correlated in time with one another. (e) Intensity of the peaks *R*_6_^3^ and *R*_9_^2^ as a function of time. The almost constant sum of the intensity confirms the correlation among these states.

The analysis of the time-dependent spectrum of sample 2 is reported in Figure 4. Figure 4a shows that the dynamics of this sample are more complex than that of sample 1, manifesting larger spectral wandering and complex jumps involving several energetic states. The richer spectral dynamics of sample 2 can be explained by the higher density of defects resulting from the plasma treatment. A higher defect density across samples may alter the charge distribution and enhance electromagnetic interactions affecting the nonlocal recombination processes and creating more spectral instabilities. Within this data set, we can identify at least two spectral regions whose dynamics can be explained by nonlocal recombination processes. The zoomed-in plot of Figure 4b shows a continuous jumping among two well-defined states that are associated with the state *E*_Nπ_^6^ = 1.676eV with *R*_m_^l^ = *R*_23_^0^, *R*_22_^0^. Their correlation is confirmed by their time-dependent intensity traces (Figure 4c). Note that in this case, the dynamic is ascribed to purely in-plane transitions among two defective sites at a similar radial distance. Additionally, we observe in Figure 4d a similar behavior with another pair of states corresponding to *R*_m_^l^ = *R*_9_^2^, *R*_6_^3^ in a region of the spectrum highlighted with the blue box in Figure 4a. It should be noted that in this region of the spectrum, emission lines could in principle be matched to multiple DAP states present in the proximity (±4 meV) of 1.96 eV. However, *R*_9_^2^, *R*_6_^3^ are the closest in energy to the experimental lines (more details in Supporting Information note 6). Due to the slow time resolution of our measurements, we cannot exclude that what appears as spectral wandering is the result from fast jumping between other states ascribed present within the line width of the transitions.

Our experimental results suggest some scenarios about the physical reasons behind the dynamic switching from different shells. Quantum emission in defective hBN can occur through the formation of Coulomb pairs among delocalized defects whose elementary electronic states follow the harmonic states of the N-π* orbitals, giving rise to peaks at specific energies in the PL spectrum. Spectral jumping is the result of the breaking of such pairs and the formation of new pairs between different lattice sites. The change in the defect distance alters the Coulomb interaction energy, generating a discrete spectral shift. The combined measurements of the spectral emission lines and their dynamic spectral shifts could, in principle, be used to reconstruct the spatial distribution of defects involved in the nonlocal recombination process. The formation and breaking of the pairs can be tentatively attributed to two main factors: the geometry of the defects within the hBN matrix and the electromagnetic and dielectric environment. The former is the spatial arrangement of defects within the lattice, is governed by the stability of the defect clusters, and dictates the overlap between the wave functions of defective states in the formation of the Coulomb pairs. Perturbations of the electromagnetic environment can generate the decoupling of the defect sites and the consequent coupling of new pairs. Future works should focus on precise dynamical control of the electromagnetic environment, i.e., with applied electric fields, to stabilize shells and possibly to dynamically tune the single-photon emission by directing Coulomb interactions. Moreover, the use of hBN samples with a lower local or global density of defects can already result in the formation of a single stable pair, in which the spectral jumps are inhibited by the lack of possible alternatives for the recombination process, such as the regions of our sample measured in Figure 1b,f. It is important to note that the ZPLs observed in these spectra are ascribed to the transition (*E*_Nπ_^7^, *R*_5_^5^) and (*E*_Nπ_^6^, *R*_5_^0^) for samples 1 and 2, respectively, corroborating that delocalized recombination processes, give rise to quantum emission. Experiments conducted on hBN samples with lower density of defects could also reveal interesting insights regarding the correlation of the polarization of the single photon emission and the direction of the dipole moment of the Coulomb pairs.^59^ In the presence of a high density of defects, polarization-dependent measurements are challenging due to the high degeneracy of the Coulomb pairs and the large variation of the orientation of their transition dipoles.^60^ Furthermore, ultrafast spectroscopy techniques should be used to acquire a more comprehensive picture of the charge recombination dynamics, including the formation and breaking of Coulomb pairs by measuring the variations in the hBN spectral emission at time scales comparable to the radiative lifetime of the emitters.^61^ Such measurements could help reveal the connection between the time scale of the instabilities, the size, and radiative lifetime of the pairs responsible for the single photon emission in hBN.

## Conclusions

In conclusion, we have investigated the dynamics of the spectral emission of defective hBN from both as grown and argon plasma treated defective hBN on a time scale much longer than the radiative time of the emitters. We have used a phenomenological model that considers the recent discovery of harmonic states stemming from the N-π* orbitals and their nonlocal optical recombination to account for the rich spectrum of hBN emitters. Within this framework, we have shown that spectral jumps can be ascribed to the dynamical formation and breaking of pairs in a DAP-like process, leading to discrete energy shifts in the defect emission. These findings pave the way toward a rigorous control of the spectral dynamics of hBN and its use in practical applications requiring spectral stability and longer coherent times. Such control would open the possibility of using such states for quantum sensing and computation due to the long-range nature of their interactions stemming from the large dipole moment of the quantum emitters.^59^

## Supporting Information Available

Elementary excitations of quantum emitters in defective hBN, nonlocal recombination processes in defective hBN, donor–acceptor pair fitting algorithm, additional details on lattice site notation, additional details on DAP process and peak identifications, additional details on the identification of spectral jumps in sample 1, additional details on the identification of phonon replicas, additional details on the identification of spectral jumps in sample 2, and energy tables for DAP transitions in samples 1 and 2 (PDF)

## References

[ref1] Aharonovich I.; Englund D.; Toth M. Solid-State Single-Photon Emitters. Nat. Photonics 2016, 10 (10), 631–641. 10.1038/nphoton.2016.186.

[ref2] Wolfowicz G.; Heremans F. J.; Anderson C. P.; Kanai S.; Seo H.; Gali A.; Galli G.; Awschalom D. D. Quantum Guidelines for Solid-State Spin Defects. Nat. Rev. Mater. 2021, 6 (10), 906–925. 10.1038/s41578-021-00306-y.

[ref3] Schrinner P. P. J.; Olthaus J.; Reiter D. E.; Schuck C. Integration of Diamond-Based Quantum Emitters with Nanophotonic Circuits. Nano Lett. 2020, 20 (11), 8170–8177. 10.1021/acs.nanolett.0c03262. 33136413

[ref4] Spencer L.; Horder J.; Kim S.; Toth M.; Aharonovich I. Monolithic Integration of Single Quantum Emitters in hBN Bullseye Cavities. ACS Photonics 2023, 10 (12), 4417–4424. 10.1021/acsphotonics.3c01282.

[ref5] Kerski J.; Lochner P.; Ludwig A.; Wieck A. D.; Kurzmann A.; Lorke A.; Geller M. Quantum Sensor for Nanoscale Defect Characterization. Phys. Rev. Appl. 2021, 15 (2), 024029 10.1103/PhysRevApplied.15.024029.

[ref6] Gao X.; Jiang B.; Llacsahuanga Allcca A. E.; Shen K.; Sadi M. A.; Solanki A. B.; Ju P.; Xu Z.; Upadhyaya P.; Chen Y. P.; et al. High-Contrast Plasmonic-Enhanced Shallow Spin Defects in Hexagonal Boron Nitride for Quantum Sensing. Nano Lett. 2021, 21 (18), 7708–7714. 10.1021/acs.nanolett.1c02495. 34473524

[ref7] Altman E.; Brown K. R.; Carleo G.; Carr L. D.; Demler E.; Chin C.; DeMarco B.; Economou S. E.; Eriksson M. A.; Fu K.-M. C.; et al. Quantum Simulators: Architectures and Opportunities. PRX Quantum 2021, 2 (1), 017003 10.1103/PRXQuantum.2.017003.

[ref8] Seo H.; Ma H.; Govoni M.; Galli G. Designing Defect-Based Qubit Candidates in Wide-Gap Binary Semiconductors for Solid-State Quantum Technologies. Phys. Rev. Mater. 2017, 1 (7), 075002 10.1103/PhysRevMaterials.1.075002.

[ref9] Huber D.; Reindl M.; Huo Y.; Huang H.; Wildmann J. S.; Schmidt O. G.; Rastelli A.; Trotta R. Highly Indistinguishable and Strongly Entangled Photons from Symmetric GaAs Quantum Dots. Nat. Commun. 2017, 8 (1), 15506 10.1038/ncomms15506. 28548081 PMC5458553

[ref10] Spokoyny B.; Utzat H.; Moon H.; Grosso G.; Englund D.; Bawendi M. G. Effect of Spectral Diffusion on the Coherence Properties of a Single Quantum Emitter in Hexagonal Boron Nitride. J. Phys. Chem. Lett. 2020, 11 (4), 1330–1335. 10.1021/acs.jpclett.9b02863. 32017564

[ref11] Wang J.; Sciarrino F.; Laing A.; Thompson M. G. Integrated Photonic Quantum Technologies. Nat. Photonics 2020, 14 (5), 273–284. 10.1038/s41566-019-0532-1.

[ref12] Awschalom D. D.; Hanson R.; Wrachtrup J.; Zhou B. B. Quantum Technologies with Optically Interfaced Solid-State Spins. Nat. Photonics 2018, 12 (9), 516–527. 10.1038/s41566-018-0232-2.

[ref13] Jahnke K. D.; Sipahigil A.; Binder J. M.; Doherty M. W.; Metsch M.; Rogers L. J.; Manson N. B.; Lukin M. D.; Jelezko F. Electron-Phonon Processes of the Silicon-Vacancy Centre in Diamond. New J. Phys. 2015, 17 (4), 043011 10.1088/1367-2630/17/4/043011.

[ref14] Böttger T.; Thiel C. W.; Sun Y.; Cone R. L. Optical Decoherence and Spectral Diffusion at $1.5 μm in Er^3+^: Y_2_SiO_5_ versus Magnetic Field, Temperature, and Er^3+^ Concentration. Phys. Rev. B 2006, 73 (7), 075101 10.1103/PhysRevB.73.075101.

[ref15] Nagy R.; Niethammer M.; Widmann M.; Chen Y.-C.; Udvarhelyi P.; Bonato C.; Hassan J. U.; Karhu R.; Ivanov I. G.; Son N. T.; et al. High-Fidelity Spin and Optical Control of Single Silicon-Vacancy Centres in Silicon Carbide. Nat. Commun. 2019, 10 (1), 1954 10.1038/s41467-019-09873-9. 31028260 PMC6486615

[ref16] Sangtawesin S.; Dwyer B. L.; Srinivasan S.; Allred J. J.; Rodgers L. V. H.; De Greve K.; Stacey A.; Dontschuk N.; O’Donnell K. M.; Hu D.; et al. Origins of Diamond Surface Noise Probed by Correlating Single-Spin Measurements with Surface Spectroscopy. Phys. Rev. X 2019, 9 (3), 031052 10.1103/PhysRevX.9.031052.

[ref17] Tonndorf P.; Schmidt R.; Schneider R.; Kern J.; Buscema M.; Steele G. A.; Castellanos-Gomez A.; van der Zant H. S. J.; Michaelis de Vasconcellos S.; Bratschitsch R. Single-Photon Emission from Localized Excitons in an Atomically Thin Semiconductor. Optica 2015, 2 (4), 347–352. 10.1364/OPTICA.2.000347.

[ref18] Conradt F.; Bezold V.; Wiechert V.; Huber S.; Mecking S.; Leitenstorfer A.; Tenne R. Electric-Field Fluctuations as the Cause of Spectral Instabilities in Colloidal Quantum Dots. Nano Lett. 2023, 23 (21), 9753–9759. 10.1021/acs.nanolett.3c02318. 37871158 PMC10636921

[ref19] Fournier C.; Watanabe K.; Taniguchi T.; Barjon J.; Buil S.; Hermier J.-P.; Delteil A. Investigating the Fast Spectral Diffusion of a Quantum Emitter in hBN Using Resonant Excitation and Photon Correlations. Phys. Rev. B 2023, 107 (19), 195304 10.1103/PhysRevB.107.195304.

[ref20] White S.; Stewart C.; Solntsev A. S.; Li C.; Toth M.; Kianinia M.; Aharonovich I. Phonon Dephasing and Spectral Diffusion of Quantum Emitters in Hexagonal Boron Nitride. Optica 2021, 8 (9), 1153–1158. 10.1364/OPTICA.431262.

[ref21] Rabouw F. T.; Kamp M.; van Dijk-Moes R. J. A.; Gamelin D. R.; Koenderink A. F.; Meijerink A.; Vanmaekelbergh D. Delayed Exciton Emission and Its Relation to Blinking in CdSe Quantum Dots. Nano Lett. 2015, 15 (11), 7718–7725. 10.1021/acs.nanolett.5b03818. 26496661

[ref22] Fernée M. J.; Plakhotnik T.; Louyer Y.; Littleton B. N.; Potzner C.; Tamarat P.; Mulvaney P.; Lounis B. Spontaneous Spectral Diffusion in CdSe Quantum Dots. J. Phys. Chem. Lett. 2012, 3 (12), 1716–1720. 10.1021/jz300456h. 26285734

[ref23] Mu Z.; Zhou Y.; Chen D.; Fröch J. E.; Yang J.; Li X.; Aharonovich I.; Gao W.-B. Observation of Binary Spectral Jumps in Color Centers in Diamond. Adv. Opt. Mater. 2020, 8 (19), 2000495 10.1002/adom.202000495.

[ref24] Fotso H. F.; Feiguin A. E.; Awschalom D. D.; Dobrovitski V. V. Suppressing Spectral Diffusion of Emitted Photons with Optical Pulses. Phys. Rev. Lett. 2016, 116 (3), 033603 10.1103/PhysRevLett.116.033603. 26849596

[ref25] Tran T. T.; Bradac C.; Solntsev A. S.; Toth M.; Aharonovich I. Suppression of Spectral Diffusion by Anti-Stokes Excitation of Quantum Emitters in Hexagonal Boron Nitride. Appl. Phys. Lett. 2019, 115 (7), 071102 10.1063/1.5099631.

[ref26] Walden-Newman W.; Sarpkaya I.; Strauf S. Quantum Light Signatures and Nanosecond Spectral Diffusion from Cavity-Embedded Carbon Nanotubes. Nano Lett. 2012, 12 (4), 1934–1941. 10.1021/nl204402v. 22439967

[ref27] Daveau R. S.; Vandekerckhove T.; Mukherjee A.; Wang Z.; Shan J.; Mak K. F.; Vamivakas A. N.; Fuchs G. D. Spectral and Spatial Isolation of Single Tungsten Diselenide Quantum Emitters Using Hexagonal Boron Nitride Wrinkles. APL Photonics 2020, 5 (9), 096105 10.1063/5.0013825.

[ref28] Basso Basset F.; Bietti S.; Reindl M.; Esposito L.; Fedorov A.; Huber D.; Rastelli A.; Bonera E.; Trotta R.; Sanguinetti S. High-Yield Fabrication of Entangled Photon Emitters for Hybrid Quantum Networking Using High-Temperature Droplet Epitaxy. Nano Lett. 2018, 18 (1), 505–512. 10.1021/acs.nanolett.7b04472. 29239186

[ref29] Li X.; Shepard G. D.; Cupo A.; Camporeale N.; Shayan K.; Luo Y.; Meunier V.; Strauf S. Nonmagnetic Quantum Emitters in Boron Nitride with Ultranarrow and Sideband-Free Emission Spectra. ACS Nano 2017, 11 (7), 6652–6660. 10.1021/acsnano.7b00638. 28521091

[ref30] Lyu C.; Zhu Y.; Gu P.; Qiao J.; Watanabe K.; Taniguchi T.; Ye Y. Single-Photon Emission from Two-Dimensional Hexagonal Boron Nitride Annealed in a Carbon-Rich Environment. Appl. Phys. Lett. 2020, 117 (24), 244002 10.1063/5.0025792.

[ref31] Tran T. T.; Elbadawi C.; Totonjian D.; Lobo C. J.; Grosso G.; Moon H.; Englund D. R.; Ford M. J.; Aharonovich I.; Toth M. Robust Multicolor Single Photon Emission from Point Defects in Hexagonal Boron Nitride. ACS Nano 2016, 10 (8), 7331–7338. 10.1021/acsnano.6b03602. 27399936

[ref32] Tran T. T.; Bray K.; Ford M. J.; Toth M.; Aharonovich I. Quantum Emission from Hexagonal Boron Nitride Monolayers. Nat. Nanotechnol. 2016, 11 (1), 37–41. 10.1038/nnano.2015.242. 26501751

[ref33] Grosso G.; Moon H.; Lienhard B.; Ali S.; Efetov D. K.; Furchi M. M.; Jarillo-Herrero P.; Ford M. J.; Aharonovich I.; Englund D. Tunable and High-Purity Room Temperature Single-Photon Emission from Atomic Defects in Hexagonal Boron Nitride. Nat. Commun. 2017, 8 (1), 705 10.1038/s41467-017-00810-2. 28951591 PMC5615041

[ref34] Stern H. L.; Gu Q.; Jarman J.; Eizagirre Barker S.; Mendelson N.; Chugh D.; Schott S.; Tan H. H.; Sirringhaus H.; Aharonovich I.; Atatüre M. Room-Temperature Optically Detected Magnetic Resonance of Single Defects in Hexagonal Boron Nitride. Nat. Commun. 2022, 13 (1), 618 10.1038/s41467-022-28169-z. 35105864 PMC8807746

[ref35] Gottscholl A.; Kianinia M.; Soltamov V.; Orlinskii S.; Mamin G.; Bradac C.; Kasper C.; Krambrock K.; Sperlich A.; Toth M.; Aharonovich I.; Dyakonov V. Initialization and Read-out of Intrinsic Spin Defects in a van Der Waals Crystal at Room Temperature. Nat. Mater. 2020, 19 (5), 540–545. 10.1038/s41563-020-0619-6. 32094496

[ref36] Exarhos A. L.; Hopper D. A.; Patel R. N.; Doherty M. W.; Bassett L. C. Magnetic-Field-Dependent Quantum Emission in Hexagonal Boron Nitride at Room Temperature. Nat. Commun. 2019, 10 (1), 222 10.1038/s41467-018-08185-8. 30644413 PMC6333818

[ref37] Haykal A.; Tanos R.; Minotto N.; Durand A.; Fabre F.; Li J.; Edgar J. H.; Ivády V.; Gali A.; Michel T.; Dréau A.; Gil B.; Cassabois G.; Jacques V. Decoherence of V_B_^–^ spin Defects in Monoisotopic Hexagonal Boron Nitride. Nat. Commun. 2022, 13 (1), 4347 10.1038/s41467-022-31743-0. 35896526 PMC9329290

[ref38] Ramsay A. J.; Hekmati R.; Patrickson C. J.; Baber S.; Arvidsson-Shukur D. R. M.; Bennett A. J.; Luxmoore I. J. Coherence Protection of Spin Qubits in Hexagonal Boron Nitride. Nat. Commun. 2023, 14 (1), 461 10.1038/s41467-023-36196-7. 36709208 PMC9884286

[ref39] Gottscholl A.; Diez M.; Soltamov V.; Kasper C.; Sperlich A.; Kianinia M.; Bradac C.; Aharonovich I.; Dyakonov V. Room Temperature Coherent Control of Spin Defects in Hexagonal Boron Nitride. Sci. Adv. 2021, 7 (14), eabf3630 10.1126/sciadv.abf3630. 33811078 PMC11059373

[ref40] Wolters J.; Sadzak N.; Schell A. W.; Schröder T.; Benson O. Measurement of the Ultrafast Spectral Diffusion of the Optical Transition of Nitrogen Vacancy Centers in Nano-Size Diamond Using Correlation Interferometry. Phys. Rev. Lett. 2013, 110 (2), 027401 10.1103/PhysRevLett.110.027401. 23383937

[ref41] Orphal-Kobin L.; Unterguggenberger K.; Pregnolato T.; Kemf N.; Matalla M.; Unger R.-S.; Ostermay I.; Pieplow G.; Schröder T. Optically Coherent Nitrogen-Vacancy Defect Centers in Diamond Nanostructures. Phys. Rev. X 2023, 13 (1), 011042 10.1103/PhysRevX.13.011042.

[ref42] Martin Z. O.; Senichev A.; Peana S.; Lawrie B. J.; Lagutchev A. S.; Boltasseva A.; Shalaev V. M. Photophysics of Intrinsic Single-Photon Emitters in Silicon Nitride at Low Temperatures. Advanced Quantum Technologies 2023, 6 (11), 2300099 10.1002/qute.202300099.

[ref43] Neuhauser R. G.; Shimizu K. T.; Woo W. K.; Empedocles S. A.; Bawendi M. G. Correlation between Fluorescence Intermittency and Spectral Diffusion in Single Semiconductor Quantum Dots. Phys. Rev. Lett. 2000, 85 (15), 3301–3304. 10.1103/PhysRevLett.85.3301. 11019326

[ref44] Hofmann M. S.; Glückert J. T.; Noé J.; Bourjau C.; Dehmel R.; Högele A. Bright, Long-Lived and Coherent Excitons in Carbon Nanotube Quantum Dots. Nat. Nanotechnol. 2013, 8 (7), 502–505. 10.1038/nnano.2013.119. 23812185

[ref45] Peterson J. J.; Krauss T. D. Fluorescence Spectroscopy of Single Lead Sulfide Quantum Dots. Nano Lett. 2006, 6 (3), 510–514. 10.1021/nl0525756. 16522053

[ref46] Mendelson N.; Chugh D.; Reimers J. R.; Cheng T. S.; Gottscholl A.; Long H.; Mellor C. J.; Zettl A.; Dyakonov V.; Beton P. H.; et al. Identifying Carbon as the Source of Visible Single-Photon Emission from Hexagonal Boron Nitride. Nat. Mater. 2021, 20 (3), 321–328. 10.1038/s41563-020-00850-y. 33139892

[ref47] Pelliciari J.; Mejia E.; Woods J. M.; Gu Y.; Li J.; Chand S. B.; Fan S.; Watanabe K.; Taniguchi T.; Bisogni V.; Grosso G. Elementary Excitations of Single-Photon Emitters in Hexagonal Boron Nitride. Nat. Mater. 2024, 23 (9), 1230–1236. 10.1038/s41563-024-01866-4. 38654140

[ref48] Li S.; Pershin A.; Thiering G.; Udvarhelyi P.; Gali A. Ultraviolet Quantum Emitters in Hexagonal Boron Nitride from Carbon Clusters. J. Phys. Chem. Lett. 2022, 13 (14), 3150–3157. 10.1021/acs.jpclett.2c00665. 35362989 PMC9014460

[ref49] Hayee F.; Yu L.; Zhang J. L.; Ciccarino C. J.; Nguyen M.; Marshall A. F.; Aharonovich I.; Vučković J.; Narang P.; Heinz T. F.; Dionne J. A. Revealing Multiple Classes of Stable Quantum Emitters in Hexagonal Boron Nitride with Correlated Optical and Electron Microscopy. Nat. Mater. 2020, 19 (5), 534–539. 10.1038/s41563-020-0616-9. 32094492

[ref50] Reimers J. R.; Shen J.; Kianinia M.; Bradac C.; Aharonovich I.; Ford M. J.; Piecuch P. Photoluminescence, Photophysics, and Photochemistry of the V_B_^–^ Defect in Hexagonal Boron Nitride. Phys. Rev. B 2020, 102 (14), 144105 10.1103/PhysRevB.102.144105.

[ref51] Li S.; Gali A. Identification of an Oxygen Defect in Hexagonal Boron Nitride. J. Phys. Chem. Lett. 2022, 13 (41), 9544–9551. 10.1021/acs.jpclett.2c02687. 36201340 PMC9589898

[ref52] Tan Q.; Lai J.-M.; Liu X.-L.; Guo D.; Xue Y.; Dou X.; Sun B.-Q.; Deng H.-X.; Tan P.-H.; Aharonovich I.; Gao W.; Zhang J. Donor-Acceptor Pair Quantum Emitters in Hexagonal Boron Nitride. Nano Lett. 2022, 22 (3), 1331–1337. 10.1021/acs.nanolett.1c04647. 35073101

[ref53] Watanabe K.; Taniguchi T.; Kanda H. Direct-Bandgap Properties and Evidence for Ultraviolet Lasing of Hexagonal Boron Nitride Single Crystal. Nat. Mater. 2004, 3 (6), 404–409. 10.1038/nmat1134. 15156198

[ref54] Xu Z.-Q.; Elbadawi C.; Tran T. T.; Kianinia M.; Li X.; Liu D.; Hoffman T. B.; Nguyen M.; Kim S.; Edgar J. H.; Wu X.; Song L.; Ali S.; Ford M.; Toth M.; Aharonovich I. Single Photon Emission from Plasma Treated 2D Hexagonal Boron Nitride. Nanoscale 2018, 10 (17), 7957–7965. 10.1039/C7NR08222C. 29682653

[ref55] Mejia E.; Woods J. M.; Chand S. B.; Ramjattan E.; Taniguchi T.; Watanabe K.; Pelliciari J.; Grosso G. General Algorithm for Characterization of Donor-Acceptor Pair Recombination Processes in Solid-State Materials. Opt. Mater. Express 2024, 14 (9), 2122–2133. 10.1364/OME.529240.

[ref56] Dean P. J.; Henry C. H.; Frosch C. J. Infrared Donor-Acceptor Pair Spectra Involving the Deep Oxygen Donor in Gallium Phosphide. Phys. Rev. 1968, 168 (3), 812–816. 10.1103/PhysRev.168.812.

[ref57] Laturia A.; Van de Put M. L.; Vandenberghe W. G. Dielectric Properties of Hexagonal Boron Nitride and Transition Metal Dichalcogenides: From Monolayer to Bulk. npj 2D Materials and Applications 2018, 2 (1), 6 10.1038/s41699-018-0050-x.

[ref58] Shotan Z.; Jayakumar H.; Considine C. R.; Mackoit M.; Fedder H.; Wrachtrup J.; Alkauskas A.; Doherty M. W.; Menon V. M.; Meriles C. A. Photoinduced Modification of Single-Photon Emitters in Hexagonal Boron Nitride. ACS Photonics 2016, 3 (12), 2490–2496. 10.1021/acsphotonics.6b00736.

[ref59] Bilgin A.; Hammock I.; Estes J.; Jin Y.; Bernien H.; High A.; Galli G. Donor-Acceptor Pairs in Wide-Bandgap Semiconductors for Quantum Technology Applications. npj Comput. Mater. 2024, 10, 7 10.1038/s41524-023-01190-6.

[ref60] Henry C. H.; Faulkner R. A.; Nassau K. Donor-Acceptor Pair Lines in Cadmium Sulfide. Phys. Rev. 1969, 183 (3), 798–806. 10.1103/PhysRev.183.798.

[ref61] Niladari Raju M. V.; Mohanty M. E.; Bangal P. R.; Vaidya J. R. Synthesis and Ultrafast Dynamics of a Donor-Acceptor-Donor Molecule Having Optoelectronic Properties. J. Phys. Chem. C 2015, 119 (16), 8563–8575. 10.1021/acs.jpcc.5b02063.

