# Supplementary material for: Dynamic Interplay of Nonlocal Recombination Pathways in Quantum Emitters in Hexagonal Boron Nitride
Source: J Phys Chem C Nanomater Interfaces. 2025 Jan 16;129(4):2044–53. doi: 10.1021/acs.jpcc.4c07147 (PMC11789140; doi:10.1021/acs.jpcc.4c07147)
Supplement: Supplementary file 1 — jp4c07147_si_001.pdf [file jp4c07147_si_001.pdf]

SUPPLEMENTARY INFORMATION

Dynamic Interplay of Nonlocal Recombination Pathways in Quantum Emitters in Hexagonal Boron Nitride

Enrique A. Mejia,[1] John M. Woods,[1] Ashok Adhikari[1], Charanjot Singh[1], Takashi Taniguchi,[2] Kenji Watanabe,[2] Valentina Bisogni,[3] Zdeněk Sofer,[4] Jonathan Pelliciari,[3] and Gabriele Grosso[1,5,*]

1. *Photonics Initiative, Advanced Science Research Center, City University of New York, New York, NY 10031, USA*
2. *Research Center for Electronic and Optical Materials, National Institute for Materials Science, 1-1 Namiki, Tsukuba 305-0044, Japan*
3. *National Synchrotron Light Source II, Brookhaven National Laboratory, Upton, NY 11973, USA*
4. *Department of Inorganic Chemistry, University of Chemistry and Technology Prague, Technická 5, 166 28, Prague 6, Czech Republic*
5. *Physics Program, Graduate Center, City University of New York, New York, NY, 10016, USA*

* ggrosso@gc.cuny.edu

Supplementary Note 1: Elementary excitations of quantum emitters in defective hBN

In Ref. [1], we combined the harmonic states of the N-π^* orbitals with the donor acceptor pair (DAP) process to develop a phenomenological model for quantum emitters (QEs) in the visible part of the spectrum of hBN. Harmonic energy levels were uncovered by probing the N-π^* orbitals near the N K edge of defective hBN through resonant inelastic X-Ray scattering (RIXS). In RIXS processes, core electrons are resonantly excited to the conduction band to create short-lived intermediate states which recombine radiatively. For these experiments, we used samples of high-quality pristine hBN and defective hBN treated with argon-plasma. When RIXS is performed on at incident energies corresponding to transitions between N 1s and N π*, we do not observe any non-trivial signal in the RIXS spectrum of pristine hBN. However, when highly defective hBN is probed, we observe a series of equally spaced peaks in the RIXS spectrum wextending up 2.30 eV. This sequence of harmonic lines appears in all measurements performed on highly defective hBN of various thicknesses when probed along the N π* transition energy. The harmonic peaks have a constant width (FWHM ~ 20meV that corresponds to the instrumental broadening of our detector). The elementary energy for such harmonics uncovered in the RIXS spectrum was found to be $\mathrm{E}^{0}_{\mathrm{N}\pi} \simeq$ 285 meV, and we denote harmonics using the notation $\mathrm{E}^{\mathrm{n}}_{\mathrm{N}\pi} \simeq \mathrm{n}\ \mathrm{E}^{0}_{\mathrm{N}\pi}$. It should additionally be noted that the intensity of these peaks cannot only be ascribed to simply phonon transitions as the intensity of such peaks in relation to their harmonic number is non-exponential and non-monotonic.

Supplementary Note 2: Nonlocal recombination processes in defective hBN.

A standard DAP series can be described using the following equation:

$$\{\mathrm{E}_{\mathrm{DAP}}\} = \{\mathrm{E}_{\mathrm{DA}} + \frac{\mathrm{e}^2}{4\pi\,|\epsilon\cdot\vec{R}_d|} : \mathrm{d} \in \mathrm{N}\} \tag{S1}$$

In which $\mathrm{E}_{\mathrm{DA}} = |\mathrm{E}_{\mathrm{D}} - \mathrm{E}_{\mathrm{A}}|$, where E_{D} and E_{A} are the donor and acceptor energy levels respectively, $\epsilon = \epsilon_0\epsilon_{\mathrm{hBN}}$ is the permittivity of the hBN system, and $|\vec{R}_d|$ is an ordered set of lattice site distances present in hBN, which can generally be described by the following sequence:

$$\{R_d\} = \{|i\vec{a} + j\vec{b} + k\,\vec{c} + l\vec{\delta}| : i, j, k \in N, l = 0,1\} \qquad (S2)$$

To compare our spectral data to a donor acceptor pair model, we employ the fitting code described in Supplementary Note 3. In our previous works[1,2], we found that the spectrum of highly defective hBN hosts several values of the fundamental DAP transition (E_{DA}) and their values are remarkably similar to the those of the harmonic states $E^5_{N\pi}$, $E^6_{N\pi}$, $E^7_{N\pi}$, and $E^8_{N\pi}$ observed in RIXS measurements. In Ref. [1], we showed that these similarities are not simply a coincidence, but there is a strong correlation between the emission pattern of quantum emitters in hBN and the harmonic states unveiled by RIXS at the N π*orbitals. Therefore, we have proven that a large fraction of the single photon emission in highly defective samples of hBN can be described by a phenomenological model which included donor-acceptor pair like emission and elementary excitations observed in RIXS:

$$E_{SPE} = \{E^i_{DA} + \frac{e^2}{4\pi|\epsilon\cdot\overrightarrow{R_d}|} : i,d \in N\} = \{n\,E^0_{N\pi} + \frac{e^2}{4\pi|\epsilon\cdot\overrightarrow{R_d}|} : n,d \in N\} \qquad (S3)$$

Which is a modified form of donor-acceptor pair type recombination in which the term E^i_{DA} has been replaced by the harmonics of the elementary excitation found in RIXS, $nE^0_{N\pi}$.

Supplementary Note 3: Donor-Acceptor Pair Fitting Algorithm

Donor-acceptor-pair (DAP) recombination can take place in a variety of materials generating a sequence of lines in the emission spectrum.[4–13] The energy of DAP emission is determined by a few factors, namely the energy difference between donor (E_D) and acceptor (E_A) levels denoted in this work as $E_{DA} = |E_D - E_A|$, the dielectric tensor of the material ϵ, as well as the distance between donor and acceptor lattice sites (R_d). This can be expressed using Eq. S1. Without knowing the microscopic configuration of defects within the crystal lattice, predicting the set of $\{E_{DAP}\}$ lines present in the PL spectrum of a given material is challenging. To investigate DAP in hBN, we developed a general algorithm designed to detect DAP-like emission from spectral data[2]. This method takes in a set of peak energies extracted from spectral data (E_S) and compares them to a simulated DAP set (E_F). The fit process minimizes the differences between the two

sets by varying the value of E_{DA} which is used as a fitting parameter. E_S can be extracted from data using a variety of methods, including standard peak finding functions in many softwares. The list $\{E_F\}$ is generated using a given set of parameters following the equation:

$$\{E_F\} = \{E_{DAP}(E_{DA}, R_d, \epsilon)\} \tag{S4}$$

The algorithm looks for the presence of DAP sequences in the data by comparing the peak energies in E_S with the ones of ideal DAP sequences E_F. The values of $|E_S - E_F|$ for each value of the respective arrays, are calculated and the smallest value is stored into an array δ. The next smallest physically possible value is then found and stored into δ and so on. Upon populating δ, we define a parameter known as coincidence which tells us how well our data fits a donor acceptor pair model. We define coincidence C as:

$$C(\delta, W_{co}) = \#\delta : \delta < W_{co}$$

W_{co} is the coincidence window, namely the energy interval used to confirm the match between the experimental peak energy and on entry of the DAP sequence. W_{co} is an arbitrary parameter and can be adjusted to include in the fitting the experimental parameters, such as the spectral resolution and the peak broadening. In this work we set $W_{co} = 0.6$ eV. We consider a good match when coincidence is maximized in relation E_{DA}. After having identified the values of E_{DA} that correspond to local maxima in the coincidence plot, we perform a local search in the vicinity of these values by focusing on maximizing coincidence for particular values of ϵ and R_d along the E_{DA} axis. [2] We find that the fitting algorithm is robust against various types of noise, and that it can be reliably used to identify the same DAP resonances observed in other works.[1,2,14]

Supplementary Note 4: Additional details on lattice site notation.

In the notation R_m^l used in the main text, *m* corresponds to an in-plane set of distances ordered from smallest to largest, while *l* corresponds to an out-of-plane number of layer jumps. We find this notation convenient, as ordering simply by energy does not give an idea of where transitions occur spatially in a material, due to the calculation of energy being tied to an anisotropic permittivity in the case of hBN.

Supplementary Note 5: Additional details on DAP process and peak identifications

The identified transitions for the spectrum Figures 2d and 2f of the main text are shown in Figure S1. It is interesting to note that some patterns emerge in the emission peaks of both samples. In particular, we note how in sample 1, the transition R_4^0 appears for the fundamental energies $E_{N\pi}^5 = 1.424$ eV and $E_{N\pi}^6 = 1.683$ eV. This observation indicates that each Coulomb pair can recombine radiatively through different harmonic states, in this case $E_{N\pi}^5$ and $E_{N\pi}^6$.

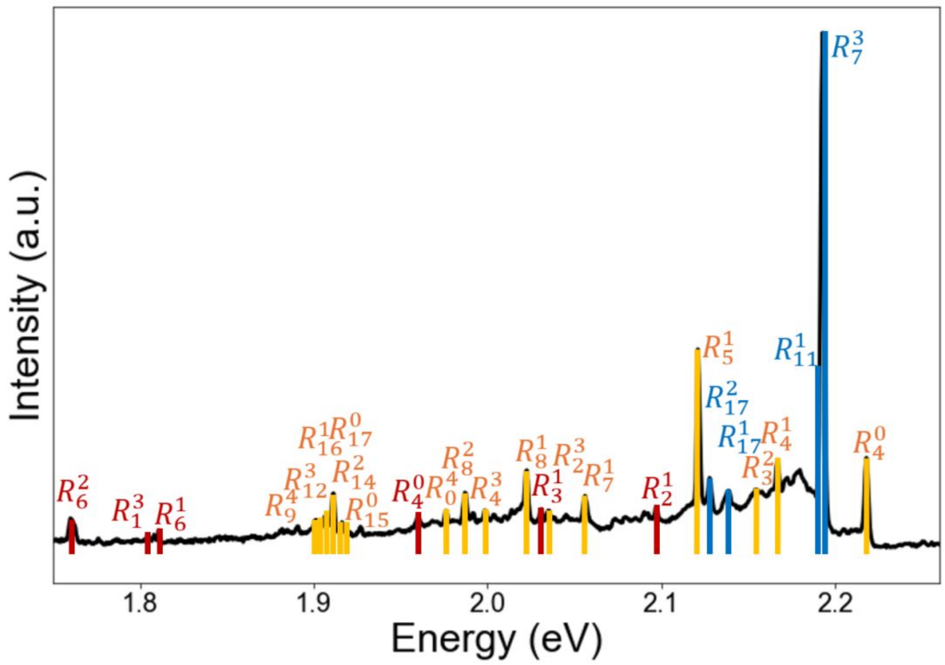

Figure S1: Overlay of the R_m^1 labels for the transitions identified in Fig. 2d of the main text.

Similarly, in Fig. S2 we observe similar recombination patterns in the overlaid composite spectrum of sample 2. In particular, we note how Coulomb pairs R_0^2 R_5^2 and R_2^3 appear in transitions corresponding to $E_{N\pi}^5 = 1.430$ eV and $E_{N\pi}^6 = 1.676$ eV. This type of emission could arise from DAP-like transitions occurring on the same defect complexes but with different fundamental emission energies.

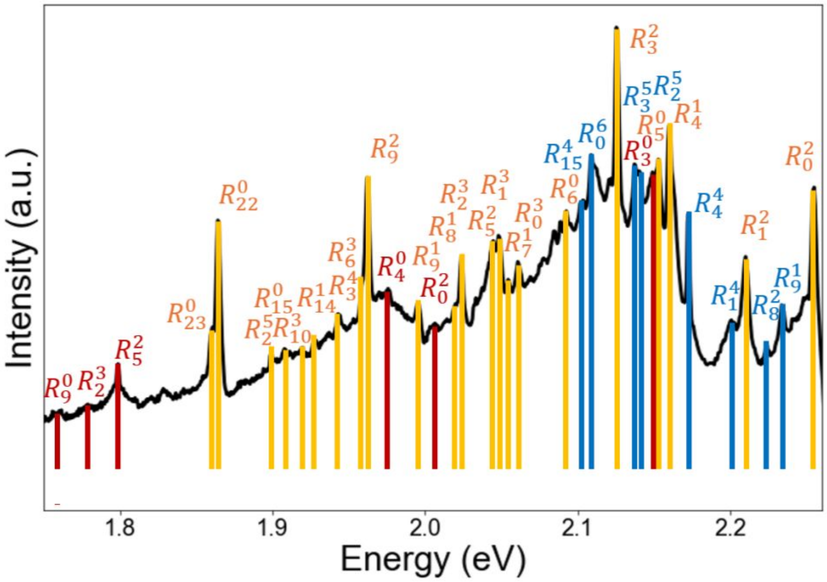

Figure S2: Overlay of the R_m^l labels for the transitions identified in Fig. 2f of the main text.

Supplemental Note 6: Additional details on the identification of spectral jumps in Sample 1.

As highlighted in Fig 3 in the main text, we observe the dynamic interplay between states associated with the fundamental energy of $E_{N\pi}^6 = 1.683$ eV. We can see that emission begins with the state associated with R_{11}^3, before turning to the state associated with R_{14}^2, then switching to R_{15}^0. Since the sum of states is almost constant (Figure S3), we can assume that these transitions are correlated, and that the recombination pathway is switching between distances at different layers and different radial distances.

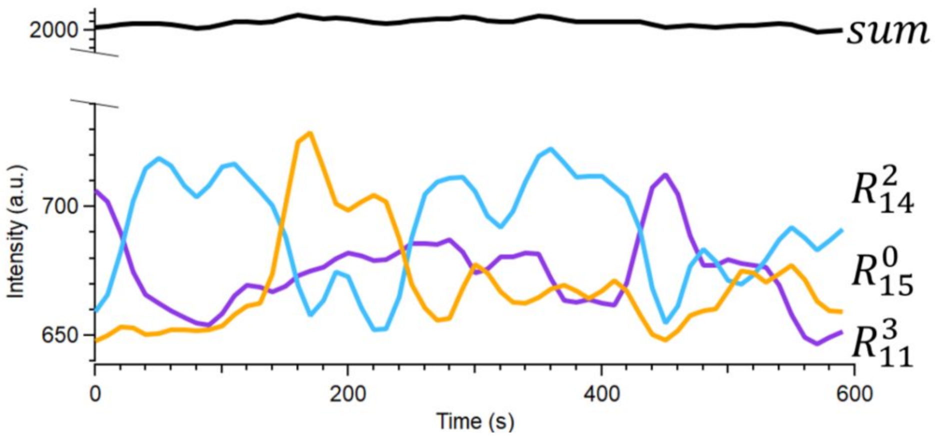

Figure S3: Dynamics of states corresponding to $E_{DA} = R_m^1 = R_{15}^0, R_{14}^2, R_{11}^3$. Note how the sum of intensities remains relatively constant while each line increases and decreases in intensity in correlated ways.

Supplemental Note 7: Additional details on the identification of phonon replicas.

It should be noted that phonon replicas are not automatically prevented from being matched under the fitting algorithm. However, phonon sidebands are usually much weaker than zero phonon lines and get hidden in the noise floor. Nevertheless, for particularly bright emitters, phonon sidebands are observed. One example is shown below where the phonon replicas of states $R_m^1 = R_7^3$ and R_{11}^1 of $E_{N\pi}^7 = 1.918$ eV appears clearly in the time-dependent spectrum of sample 1. The separation between these states is roughly 170 meV corresponding to TO phonons in hBN[15].

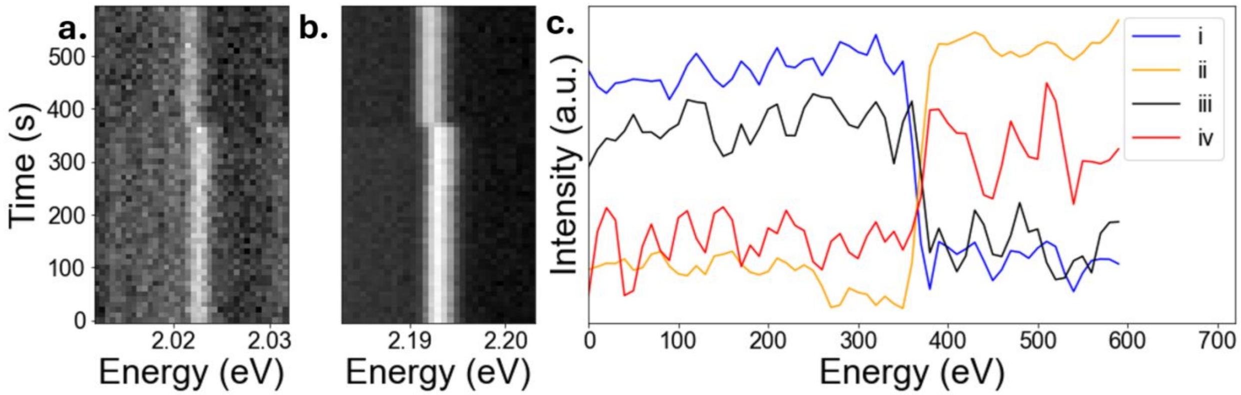

Figure S4: **a, b** - Time-dependent spectra of phonon sidebands and zero phonon lines of the transitions $R^1_m = R^3_7$ and R^1_{11}, respectively. **c** - Interplay between states attributed to transitions $R^1_m = R^3_7$ (i) and R^1_{11}(ii) with $E^7_{N\pi} = 1.918$ eV, and their phonon replicas occurring at 2.022 eV (iii) and 2.021 eV (iv). Note the correlation between traces i. and iii, suggesting a common source, and note how emission switches from i. to ii. and from iii. to iv. at around 370s.

Supplemental Note 8: Additional details on the identification of spectral jumps in sample 2.

In Figure 4 of the main text, we prove that the emission around 1.96 eV can be attributed to states corresponding to $E^6_{N\pi} = 1.676$ eV with the pairs R^3_6, and R^2_9. However, we observe significant spectral wandering of the line attributed to R^2_9. We speculate that this spectral wandering could be the result of the interplay between R^2_9 and the nearby R^0_{11} state. Since these states are within the experimental line width of each other, we cannot confirm this hypothesis in this measurement.

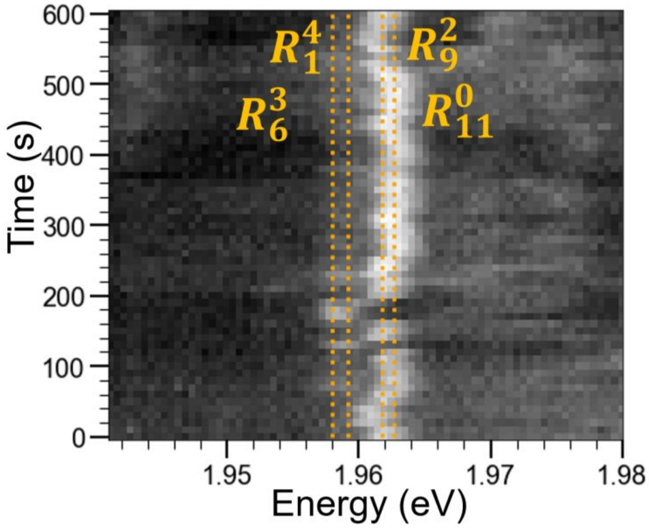

Figure S5: Spectral wandering in the time dependent spectra of sample 2. Note how line previously ascribed solely to transition at $E_{N\pi}^{6} = 1.676$ eV, $R_m^l = R_9^2$ has quite a significant amount of spectral wandering. Because the nearest line R_{11}^0 is within the linewidth of the R_9^2, the interplay of these states could be the reason for the spectral wandering.

Supplementary Note 9: Energy tables for DAP transitions in Samples 1 and 2

**d'**	**E [eV]**	**l**	**xy dist [Å]**	**m**	**z dist [Å]**
0	1.41860	1	1.455	0	0.000
1	1.17492	0	0.000	1	3.337
2	0.90487	1	1.455	1	3.337
3	0.81903	2	2.520	0	0.000
4	0.70930	3	2.910	0	0.000

5	0.67189	2	2.520	1	3.337
6	0.60723	3	2.910	1	3.337
7	0.58746	0	0.000	2	6.673
8	0.54276	1	1.455	2	6.673
9	0.53618	4	3.849	0	0.000
10	0.48779	4	3.849	1	3.337
11	0.47736	2	2.520	2	6.673
12	0.47287	5	4.365	0	0.000
13	0.45243	3	2.910	2	6.673
14	0.43867	5	4.365	1	3.337
15	0.40952	6	5.040	0	0.000
16	0.39603	4	3.849	2	6.673
17	0.39345	7	5.246	0	0.000
18	0.39164	0	0.000	3	10.010
19	0.38670	6	5.040	1	3.337
20	0.37752	1	1.455	3	10.010
21	0.37309	7	5.246	1	3.337
22	0.36836	5	4.365	2	6.673
23	0.35465	8	5.820	0	0.000
24	0.35332	2	2.520	3	10.010
25	0.34285	3	2.910	3	10.010
26	0.33952	8	5.820	1	3.337
27	0.33595	6	5.040	2	6.673
28	0.32690	7	5.246	2	6.673

29	0.32545	9	6.342	0	0.000
30	0.31626	4	3.849	3	10.010
31	0.31364	9	6.342	1	3.337
32	0.30956	10	6.667	0	0.000
33	0.30361	8	5.820	2	6.673
34	0.30162	5	4.365	3	10.010
35	0.29935	10	6.667	1	3.337
36	0.29373	0	0.000	4	13.346
37	0.28763	1	1.455	4	13.346
38	0.28468	9	6.342	2	6.673
39	0.28372	11	7.275	0	0.000
40	0.28304	6	5.040	3	10.010
41	0.27757	7	5.246	3	10.010
42	0.27649	2	2.520	4	13.346
43	0.27579	11	7.275	1	3.337
44	0.27387	10	6.667	2	6.673
45	0.27301	12	7.560	0	0.000
46	0.27138	3	2.910	4	13.346
47	0.26809	13	7.699	0	0.000
48	0.26593	12	7.560	1	3.337
49	0.26288	8	5.820	3	10.010
50	0.26137	13	7.699	1	3.337
51	0.25761	4	3.849	4	13.346
52	0.25548	11	7.275	2	6.673

53	0.25479	14	8.101	0	0.000
54	0.25031	9	6.342	3	10.010
55	0.24951	5	4.365	4	13.346
56	0.24900	14	8.101	1	3.337
57	0.24758	12	7.560	2	6.673
58	0.24389	13	7.699	2	6.673
59	0.24286	10	6.667	3	10.010
60	0.23868	6	5.040	4	13.346
61	0.23643	15	8.730	0	0.000
62	0.23537	7	5.246	4	13.346
63	0.23498	0	0.000	5	16.683
64	0.23375	14	8.101	2	6.673
65	0.23322	16	8.850	0	0.000
66	0.23182	1	1.455	5	16.683
67	0.23179	15	8.730	1	3.337
68	0.22976	11	7.275	3	10.010
69	0.22875	16	8.850	1	3.337
70	0.22716	17	9.086	0	0.000
71	0.22622	8	5.820	4	13.346
72	0.22587	2	2.520	5	16.683
73	0.22396	12	7.560	3	10.010
74	0.22306	3	2.910	5	16.683
75	0.22303	17	9.086	1	3.337
76	0.22122	13	7.699	3	10.010

77	0.21934	15	8.730	2	6.673
78	0.21805	9	6.342	4	13.346
79	0.21676	16	8.850	2	6.673
80	0.21633	18	9.541	0	0.000
81	0.21522	4	3.849	5	16.683
82	0.21357	14	8.101	3	10.010
83	0.21308	10	6.667	4	13.346
84	0.21276	18	9.541	1	3.337
85	0.21187	17	9.086	2	6.673
86	0.21043	5	4.365	5	16.683
87	0.20476	19	10.080	0	0.000
88	0.20407	11	7.275	4	13.346
89	0.20381	6	5.040	5	16.683
90	0.20301	18	9.541	2	6.673
91	0.20266	20	10.184	0	0.000
92	0.20241	15	8.730	3	10.010
93	0.20174	7	5.246	5	16.683
94	0.20172	19	10.080	1	3.337
95	0.20038	16	8.850	3	10.010
96	0.19997	12	7.560	4	13.346
97	0.19971	20	10.184	1	3.337
98	0.19801	13	7.699	4	13.346
99	0.19672	21	10.492	0	0.000
100	0.19650	17	9.086	3	10.010

101	0.19589	8	5.820	5	16.683
102	0.19582	0	0.000	6	20.019
103	0.19402	21	10.492	1	3.337
104	0.19398	1	1.455	6	20.019
105	0.19335	19	10.080	2	6.673
106	0.19247	14	8.101	4	13.346
107	0.19158	20	10.184	2	6.673
108	0.19051	9	6.342	5	16.683
109	0.19045	2	2.520	6	20.019
110	0.18937	18	9.541	3	10.010
111	0.18876	3	2.910	6	20.019
112	0.18790	22	10.984	0	0.000
113	0.18717	10	6.667	5	16.683
114	0.18654	21	10.492	2	6.673
115	0.18554	22	10.984	1	3.337
116	0.18418	15	8.730	4	13.346
117	0.18394	4	3.849	6	20.019
118	0.18265	16	8.850	4	13.346
119	0.18163	23	11.363	0	0.000
120	0.18145	19	10.080	3	10.010
121	0.18097	11	7.275	5	16.683
122	0.18092	5	4.365	6	20.019
123	0.17999	20	10.184	3	10.010
124	0.17969	17	9.086	4	13.346

125	0.17950	23	11.363	1	3.337
126	0.17897	22	10.984	2	6.673
127	0.17873	24	11.548	0	0.000
128	0.17810	12	7.560	5	16.683
129	0.17733	25	11.639	0	0.000
130	0.17671	13	7.699	5	16.683
131	0.17669	24	11.548	1	3.337
132	0.17666	6	5.040	6	20.019
133	0.17579	21	10.492	3	10.010
134	0.17534	25	11.639	1	3.337
135	0.17531	7	5.246	6	20.019
136	0.17419	18	9.541	4	13.346
137	0.17353	23	11.363	2	6.673
138	0.17331	26	11.909	0	0.000
139	0.17274	14	8.101	5	16.683
140	0.17145	26	11.909	1	3.337
141	0.17142	8	5.820	6	20.019
142	0.17099	24	11.548	2	6.673
143	0.16976	25	11.639	2	6.673
144	0.16941	22	10.984	3	10.010
145	0.16797	19	10.080	4	13.346
146	0.16785	0	0.000	7	23.356
147	0.16779	9	6.342	6	20.019
148	0.16681	20	10.184	4	13.346

149	0.16668	1	1.455	7	23.356
150	0.16667	15	8.730	5	16.683
151	0.16623	26	11.909	2	6.673
152	0.16603	27	12.431	0	0.000
153	0.16553	16	8.850	5	16.683
154	0.16549	10	6.667	6	20.019
155	0.16478	23	11.363	3	10.010
156	0.16443	2	2.520	7	23.356
157	0.16440	27	12.431	1	3.337
158	0.16381	28	12.600	0	0.000
159	0.16345	21	10.492	4	13.346
160	0.16333	3	2.910	7	23.356
161	0.16332	17	9.086	5	16.683
162	0.16272	29	12.684	0	0.000
163	0.16260	24	11.548	3	10.010
164	0.16224	28	12.600	1	3.337
165	0.16154	25	11.639	3	10.010
166	0.16119	29	12.684	1	3.337
167	0.16116	11	7.275	6	20.019
168	0.16018	4	3.849	7	23.356
169	0.15978	27	12.431	2	6.673
170	0.15961	30	12.932	0	0.000
171	0.15916	18	9.541	5	16.683
172	0.15912	12	7.560	6	20.019

173	0.15849	26	11.909	3	10.010
174	0.15828	22	10.984	4	13.346
175	0.15818	5	4.365	7	23.356
176	0.15815	30	12.932	1	3.337
177	0.15813	13	7.699	6	20.019
178	0.15779	28	12.600	2	6.673
179	0.15762	31	13.094	0	0.000
180	0.15682	29	12.684	2	6.673
181	0.15622	31	13.094	1	3.337
182	0.15531	6	5.040	7	23.356
183	0.15526	14	8.101	6	20.019
184	0.15478	32	13.335	0	0.000
185	0.15448	23	11.363	4	13.346
186	0.15438	7	5.246	7	23.356
187	0.15437	19	10.080	5	16.683
188	0.15402	30	12.932	2	6.673
189	0.15347	20	10.184	5	16.683
190	0.15346	32	13.335	1	3.337
191	0.15286	27	12.431	3	10.010

192	0.15268	24	11.548	4	13.346
193	0.15224	31	13.094	2	6.673
194	0.15181	25	11.639	4	13.346
195	0.15171	8	5.820	7	23.356
196	0.15112	28	12.600	3	10.010
197	0.15084	21	10.492	5	16.683
198	0.15081	15	8.730	6	20.019
199	0.15027	29	12.684	3	10.010

Table S1: Sample 1 DAP Sequence Table. Energies from DAP transitions arising from lattice parameters found for sample 1 (a = 2.520 Å, c = 6.673 Å, $\epsilon_{\parallel} = 6.999$, and $\epsilon_{\perp} = 3.685$). Values of individual lines can be extracted by adding energy to $E_{N\pi}^{5} = 1.424$ eV , $E_{N\pi}^{6} = 1.683$ eV, and and $E_{N\pi}^{7} = 1.916$ eV. d' corresponds to an ordered index of highest energy transitions moving towards lowest energy transitions for a given E_{DA}. l corresponds to the number of layer transitions, and m corresponds to the index of ordered in-plane distances.

d'	E [eV]	l	xy dist [Å]	m	z dist [Å]
0	1.4356	1	1.455	0	0.000
1	1.1573	0	0.000	1	3.337
2	0.9010	1	1.455	1	3.337
3	0.8289	2	2.520	0	0.000
4	0.7178	3	2.910	0	0.000
5	0.6739	2	2.520	1	3.337
6	0.6100	3	2.910	1	3.337
7	0.5787	0	0.000	2	6.673
8	0.5426	4	3.849	0	0.000
9	0.5367	1	1.455	2	6.673
10	0.4913	4	3.849	1	3.337
11	0.4785	5	4.365	0	0.000
12	0.4745	2	2.520	2	6.673
13	0.4505	3	2.910	2	6.673
14	0.4422	5	4.365	1	3.337
15	0.4144	6	5.040	0	0.000

16	0.3982	7	5.246	0	0.000
17	0.3958	4	3.849	2	6.673
18	0.3902	6	5.040	1	3.337
19	0.3858	0	0.000	3	10.010
20	0.3765	7	5.246	1	3.337
21	0.3726	1	1.455	3	10.010
22	0.3688	5	4.365	2	6.673
23	0.3589	8	5.820	0	0.000
24	0.3498	2	2.520	3	10.010
25	0.3428	8	5.820	1	3.337
26	0.3398	3	2.910	3	10.010
27	0.3369	6	5.040	2	6.673
28	0.3294	9	6.342	0	0.000
29	0.3280	7	5.246	2	6.673
30	0.3168	9	6.342	1	3.337
31	0.3144	4	3.849	3	10.010
32	0.3133	10	6.667	0	0.000
33	0.3050	8	5.820	2	6.673

34	0.3024	10	6.667	1	3.337
35	0.3003	5	4.365	3	10.010
36	0.2893	0	0.000	4	13.346
37	0.2871	11	7.275	0	0.000
38	0.2862	9	6.342	2	6.673
39	0.2836	1	1.455	4	13.346
40	0.2824	6	5.040	3	10.010
41	0.2787	11	7.275	1	3.337
42	0.2771	7	5.246	3	10.010
43	0.2763	12	7.560	0	0.000
44	0.2755	10	6.667	2	6.673
45	0.2732	2	2.520	4	13.346
46	0.2713	13	7.699	0	0.000
47	0.2687	12	7.560	1	3.337
48	0.2683	3	2.910	4	13.346
49	0.2641	13	7.699	1	3.337
50	0.2628	8	5.820	3	10.010
51	0.2579	14	8.101	0	0.000

52	0.2572	11	7.275	2	6.673
53	0.2553	4	3.849	4	13.346
54	0.2517	14	8.101	1	3.337
55	0.2505	9	6.342	3	10.010
56	0.2493	12	7.560	2	6.673
57	0.2476	5	4.365	4	13.346
58	0.2457	13	7.699	2	6.673
59	0.2432	10	6.667	3	10.010
60	0.2393	15	8.730	0	0.000
61	0.2372	6	5.040	4	13.346
62	0.2360	16	8.850	0	0.000
63	0.2355	14	8.101	2	6.673
64	0.2343	15	8.730	1	3.337
65	0.2341	7	5.246	4	13.346
66	0.2315	0	0.000	5	16.683
67	0.2313	16	8.850	1	3.337
68	0.2303	11	7.275	3	10.010
69	0.2299	17	9.086	0	0.000

70	0.2285	1	1.455	5	16.683
71	0.2255	17	9.086	1	3.337
72	0.2253	8	5.820	4	13.346
73	0.2246	12	7.560	3	10.010
74	0.2229	2	2.520	5	16.683
75	0.2219	13	7.699	3	10.010
76	0.2211	15	8.730	2	6.673
77	0.2203	3	2.910	5	16.683
78	0.2189	18	9.541	0	0.000
79	0.2185	16	8.850	2	6.673
80	0.2174	9	6.342	4	13.346
81	0.2151	18	9.541	1	3.337
82	0.2144	14	8.101	3	10.010
83	0.2136	17	9.086	2	6.673
84	0.2129	4	3.849	5	16.683
85	0.2125	10	6.667	4	13.346
86	0.2084	5	4.365	5	16.683
87	0.2072	19	10.080	0	0.000

88	0.2051	20	10.184	0	0.000
89	0.2048	18	9.541	2	6.673
90	0.2040	19	10.080	1	3.337
91	0.2038	11	7.275	4	13.346
92	0.2033	15	8.730	3	10.010
93	0.2021	6	5.040	5	16.683
94	0.2019	20	10.184	1	3.337
95	0.2013	16	8.850	3	10.010
96	0.2001	7	5.246	5	16.683
97	0.1998	12	7.560	4	13.346
98	0.1991	21	10.492	0	0.000
99	0.1979	13	7.699	4	13.346
100	0.1975	17	9.086	3	10.010
101	0.1962	21	10.492	1	3.337
102	0.1951	19	10.080	2	6.673
103	0.1945	8	5.820	5	16.683
104	0.1933	20	10.184	2	6.673
105	0.1929	0	0.000	6	20.019

106	0.1925	14	8.101	4	13.346
107	0.1912	1	1.455	6	20.019
108	0.1904	18	9.541	3	10.010
109	0.1902	22	10.984	0	0.000
110	0.1894	9	6.342	5	16.683
111	0.1883	21	10.492	2	6.673
112	0.1879	2	2.520	6	20.019
113	0.1876	22	10.984	1	3.337
114	0.1863	3	2.910	6	20.019
115	0.1862	10	6.667	5	16.683
116	0.1844	15	8.730	4	13.346
117	0.1838	23	11.363	0	0.000
118	0.1829	16	8.850	4	13.346
119	0.1825	19	10.080	3	10.010
120	0.1817	4	3.849	6	20.019
121	0.1815	23	11.363	1	3.337
122	0.1811	20	10.184	3	10.010
123	0.1809	24	11.548	0	0.000

124	0.1807	22	10.984	2	6.673
125	0.1802	11	7.275	5	16.683
126	0.1800	17	9.086	4	13.346
127	0.1794	25	11.639	0	0.000
128	0.1789	5	4.365	6	20.019
129	0.1787	24	11.548	1	3.337
130	0.1774	12	7.560	5	16.683
131	0.1773	25	11.639	1	3.337
132	0.1769	21	10.492	3	10.010
133	0.1761	13	7.699	5	16.683
134	0.1754	26	11.909	0	0.000
135	0.1752	23	11.363	2	6.673
136	0.1749	6	5.040	6	20.019
137	0.1746	18	9.541	4	13.346
138	0.1736	7	5.246	6	20.019
139	0.1734	26	11.909	1	3.337
140	0.1726	24	11.548	2	6.673
141	0.1722	14	8.101	5	16.683

142	0.1714	25	11.639	2	6.673
143	0.1706	22	10.984	3	10.010
144	0.1699	8	5.820	6	20.019
145	0.1685	19	10.080	4	13.346
146	0.1680	27	12.431	0	0.000
147	0.1678	26	11.909	2	6.673
148	0.1673	20	10.184	4	13.346
149	0.1664	9	6.342	6	20.019
150	0.1664	15	8.730	5	16.683
151	0.1663	27	12.431	1	3.337
152	0.1659	23	11.363	3	10.010
153	0.1658	28	12.600	0	0.000
154	0.1653	0	0.000	7	23.356
155	0.1653	16	8.850	5	16.683
156	0.1647	29	12.684	0	0.000
157	0.1643	10	6.667	6	20.019
158	0.1641	28	12.600	1	3.337
159	0.1640	21	10.492	4	13.346

160	0.1638	24	11.548	3	10.010
161	0.1631	17	9.086	5	16.683
162	0.1630	29	12.684	1	3.337
163	0.1627	25	11.639	3	10.010
164	0.1621	2	2.520	7	23.356
165	0.1615	30	12.932	0	0.000
166	0.1614	27	12.431	2	6.673
167	0.1611	3	2.910	7	23.356
168	0.1601	11	7.275	6	20.019
169	0.1600	30	12.932	1	3.337
170	0.1597	26	11.909	3	10.010
171	0.1595	31	13.094	0	0.000
172	0.1594	28	12.600	2	6.673
173	0.1590	18	9.541	5	16.683
174	0.1589	22	10.984	4	13.346
175	0.1584	29	12.684	2	6.673
176	0.1582	12	7.560	6	20.019
177	0.1582	4	3.849	7	23.356

178	0.1580	31	13.094	1	3.337
179	0.1572	13	7.699	6	20.019
180	0.1566	32	13.335	0	0.000
181	0.1563	5	4.365	7	23.356
182	0.1556	30	12.932	2	6.673
183	0.1552	32	13.335	1	3.337
184	0.1552	23	11.363	4	13.346
185	0.1545	14	8.101	6	20.019
186	0.1544	19	10.080	5	16.683
187	0.1540	27	12.431	3	10.010
188	0.1538	31	13.094	2	6.673
189	0.1536	6	5.040	7	23.356
190	0.1535	20	10.184	5	16.683
191	0.1534	24	11.548	4	13.346
192	0.1527	7	5.246	7	23.356
193	0.1525	25	11.639	4	13.346
194	0.1523	28	12.600	3	10.010
195	0.1515	29	12.684	3	10.010

196	0.1512	32	13.335	2	6.673
197	0.1509	21	10.492	5	16.683
198	0.1505	33	13.879	0	0.000
199	0.1502	15	8.730	6	20.019

Table S2: Sample 2 - DAP Sequence Table for sample 2.Energies from DAP transitions arising from lattice parameters found for sample 1 (a = 2.520 Å, c = 6.673 Å, $\epsilon_{\parallel} = 6.916$, and $\epsilon_{\perp} = 3.741$.). Values of individual lines can be extracted by adding energy to $E^5_{N\pi} = 1.430$ eV, $E^6_{N\pi} = 1.676$ eV and $E^7_{N\pi} = 1.918$ eV . As in table S1, d’ corresponds to an ordered index of highest energy transitions moving towards lowest energy transitions for a given E_{DA}. l corresponds to the number of layer transitions, and m corresponds to the index of ordered in-plane distances.

References:

(1) Pelliciari, J.; Mejia, E.; Woods, J. M.; Gu, Y.; Li, J.; Chand, S. B.; Fan, S.; Watanabe, K.; Taniguchi, T.; Bisogni, V.; Grosso, G. Elementary Excitations of Single-Photon Emitters in Hexagonal Boron Nitride. *Nature Materials* **2024**, *23 (9), 1230–1236.*

(2) Mejia, E.; Woods, J. M.; Chand, S. B.; Ramjattan, E.; Taniguchi, T.; Watanabe, K.; Pelliciari, J.; Grosso, G. General Algorithm for Characterization of Donor-Acceptor Pair Recombination Processes in Solid-State Materials. *Optical Materials Express*. **2024** 14(9), 2122-2133.

(3) Tran, T. T.; Elbadawi, C.; Totonjian, D.; Lobo, C. J.; Grosso, G.; Moon, H.; Englund, D. R.; Ford, M. J.; Aharonovich, I.; Toth, M. Robust Multicolor Single Photon Emission from Point Defects in Hexagonal Boron Nitride. *ACS Nano* **2016**, *10* (8), 7331–7338. https://doi.org/10.1021/acsnano.6b03602.

(4) Enck, R. C.; Honig, A. Radiative Spectra from Shallow Donor-Acceptor Electron Transfer in Silicon. *Phys. Rev.* **1969**, *177* (3), 1182–1193. https://doi.org/10.1103/PhysRev.177.1182.

(5) Abdul Fattah, T. O.; Jacobs, J.; Markevich, V. P.; Abrosimov, N. V.; Halsall, M. P.; Crowe, I. F.; Peaker, A. R. High-Resolution Photoluminescence Study on Donor-Acceptor Pair (DAP) Recombination in Silicon Crystals Co-Doped with Phosphorous and Gallium. *J. Sci. Adv. Mater. Devices* **2023**, *8* (4), 100629. https://doi.org/10.1016/j.jsamd.2023.100629.

(6) Gershenzon, M.; Trumbore, F. A.; Mikulyak, R. M.; Kowalchik, M. Radiative Recombination between Deep-Donor-Acceptor Pairs in GaP. *J. Appl. Phys.* **2004**, *36* (5), 1528–1537. https://doi.org/10.1063/1.1703082.

(7) Shrestha, N.; Grice, C. R.; Bastola, E.; Liyanage, G. K.; Phillips, A. B.; Heben, M. J.; Yan, Y.; Ellingson, R. J. Low Temperature Photoluminescence Spectroscopy of Defect and Interband Transitions in CdSexTe1-x Thin Films. *MRS Adv.* **2018**, *3* (56), 3293–3299. https://doi.org/10.1557/adv.2018.516.

(8) Krustok, J.; Mädasson, J.; Hiie, J. Photoluminescence Properties of Z-Bands in CdTe. *Phys. Status Solidi A* **1998**, *165* (2), 517–525. https://doi.org/10.1002/(SICI)1521-396X(199802)165:2<517::AID-PSSA517>3.0.CO;2-O.

(9) Ivanov, I. G.; Henry, A.; Yan, F.; Choyke, W. J.; Janzén, E. Ionization Energy of the Phosphorus Donor in 3C–SiC from the Donor-Acceptor Pair Emission. *J. Appl. Phys.* **2010**, *108* (6), 063532. https://doi.org/10.1063/1.3487480.

(10) Urban, J. M.; Nguyen, T. H. T.; Chehade, G.; Delteil, A.; Trippé-Allard, G.; Delport, G.; Deleporte, E.; Hermier, J.-P.; Garrot, D. Discrete Donor–Acceptor Pair Transitions in CH3NH3PbI3 Perovskite Single Crystals. *Phys. Status Solidi RRL – Rapid Res. Lett.* **2023**, *17* (7), 2300005. https://doi.org/10.1002/pssr.202300005.

(11) Gorji, S.; Krecmarova, M.; Molina, A.; Asensio, M. C.; Gualdrón-Reyes, A. F.; Rodríguez-Romero, J.; Pashaei-Adl, H.; Canet-Albiach, R.; Schio, L.; Tormen, M.; Floreano, L.; Mora-Seró, I.; Pastor, J. M.; Sánchez-Royo, J. F.; Matutano, G. M. Donor-Acceptor Discrete Optical Emission in 2D Perovskites. *ArXiv Cond-Matmes-Hall* **2022**.

(12) Cansu-Ergun, E. G. Chemical Insight Into Benzimidazole Containing Donor-Acceptor-Donor Type Π-Conjugated Polymers: Benzimidazole As An Acceptor. *Polym. Rev.* **2018**, *58* (1), 42–62. https://doi.org/10.1080/15583724.2017.1329210.

(13) Irfan, M.; Iqbal, J.; Sadaf, S.; Eliasson, B.; Rana, U. A.; Ud-din Khan, S.; Ayub, K. Design of Donor–Acceptor–Donor (D–A–D) Type Small Molecule Donor Materials with Efficient Photovoltaic Parameters. *Int. J. Quantum Chem.* **2017**, *117* (10), e25363. https://doi.org/10.1002/qua.25363.

(14) Tan, Q.; Lai, J.-M.; Liu, X.-L.; Guo, D.; Xue, Y.; Dou, X.; Sun, B.-Q.; Deng, H.-X.; Tan, P.-H.; Aharonovich, I.; Gao, W.; Zhang, J. Donor–Acceptor Pair Quantum Emitters in

Hexagonal Boron Nitride. *Nano Lett.* **2022**, *22* (3), 1331–1337. https://doi.org/10.1021/acs.nanolett.1c04647.
(15) Michel, K. H.; Verberck, B. Phonon Dispersions and Piezoelectricity in Bulk and Multilayers of Hexagonal Boron Nitride. *Phys. Rev. B* **2011**, *83* (11), 115328. https://doi.org/10.1103/PhysRevB.83.115328.
